# Mammalian N1-adenosine PARylation is a reversible DNA modification

**DOI:** 10.1038/s41467-022-33731-w

**Published:** 2022-10-17

**Authors:** Michael U. Musheev, Lars Schomacher, Amitava Basu, Dandan Han, Laura Krebs, Carola Scholz, Christof Niehrs

**Affiliations:** 1 Institute of Molecular Biology (IMB), 55128 Mainz, Germany; 2 Division of Molecular Embryology, DKFZ-ZMBH Alliance, 69120 Heidelberg, Germany; 3 Present Address: STEMCELL Technologies Germany GmbH, 50933 Cologne, Germany

**Keywords:** DNA, PolyADP-ribosylation, DNA repair enzymes, Mass spectrometry

## Abstract

Poly-ADP-ribosylation (PARylation) is regarded as a protein-specific modification. However, some PARPs were recently shown to modify DNA termini in vitro. Here, we use ultrasensitive mass spectrometry (LC-MS/MS), anti-PAR antibodies, and anti-PAR reagents to show that mammalian DNA is physiologically PARylated and to different levels in primary tissues. Inhibition of PAR glycohydrolase (PARG) increases DNA PARylation, supporting that the modification is reversible. DNA PARylation requires PARP1 and in vitro PARP1 PARylates single-stranded DNA, while PARG reverts the modification. DNA PARylation occurs at the N1-position of adenosine residues to form N1-Poly(ADP-ribosyl)-deoxyadenosine. Through partial hydrolysis of mammalian gDNA we identify PAR-DNA via the diagnostic deamination product N1-ribosyl-deoxyinosine to occur in vivo. The discovery of N1-adenosine PARylation as a DNA modification establishes the conceptual and methodological framework to elucidate its biological relevance and extends the role of PARP enzymes.

## Abstract (web summary)

Poly-ADP-ribosylation (PARylation) is a well-known posttranslational modification of proteins. Here the authors show that beyond proteins also mammalian single-stranded DNA is PARylated in vitro and in vivo.

## Introduction

ADP-ribosylation of proteins is a posttranslational modification where ADP-ribose from nicotinamide adenine dinucleotide (NAD^+^) is transferred to amino acid residues of target polypeptides. The modification occurs in form of mono- and poly-ADP-ribosylation (MARylation and PARylation, respectively), involving transfer of a single or multiple ADP-ribose moieties, respectively. ADP-ribosylation is catalyzed by the ADP-ribosyltransferase superfamily of enzymes that occur in all kingdoms of life and that are thought to have evolved as a defense mechanism in bacterial virus-host interactions^1^. In mammals, PARylation regulates a vast array of mostly nuclear processes like transcription, DNA replication, chromatin remodeling, RNA splicing, and notably DNA repair^2,3^. PARylation is catalyzed by poly(ADP-ribose) polymerases (PARPs), which generate long branched polymers of ADP-ribose. Among them, PARP1 accounts for most PARylation activity induced by DNA damage, and it is a therapeutic cancer target^2,3^.

Although in mammals PARylation is regarded as a protein-specific modification, in lower organisms MARylation of DNA was reported. In butterflies, mollusks and *Streptomyces*, the enzyme-toxins pierisin, CARP-1 and Scabin, respectively, can MARylate guanosine residues in DNA^4–6^. In butterflies, DNA-MARylation may potentially serve as defense factor against parasitism^7^. In *Mycobacterium*, the DarT enzyme-toxin MARylates thymidines in single-stranded (ss) DNA^8,9^. Interestingly, it was recently shown in vitro that mammalian PARP1 and PARP2 can PARylate and PARP3 can MARylate phosphorylated DNA 3′- and 5′- ends^10–14^. Thus, DNA PARylation is chemically plausible but whether mammalian DNA is physiologically PARylated, where in the genome the modification occurs, and what its physiological role might be, remains elusive.

In this study, we reveal PARP1 to PARylate adenosine residues in ssDNA and identify N1-Poly(ADP-ribosyl)-deoxyadenosine as a reversible DNA modification in mammals.

## Results

### Mammalian DNA is PARylated

**Fig. 1:**
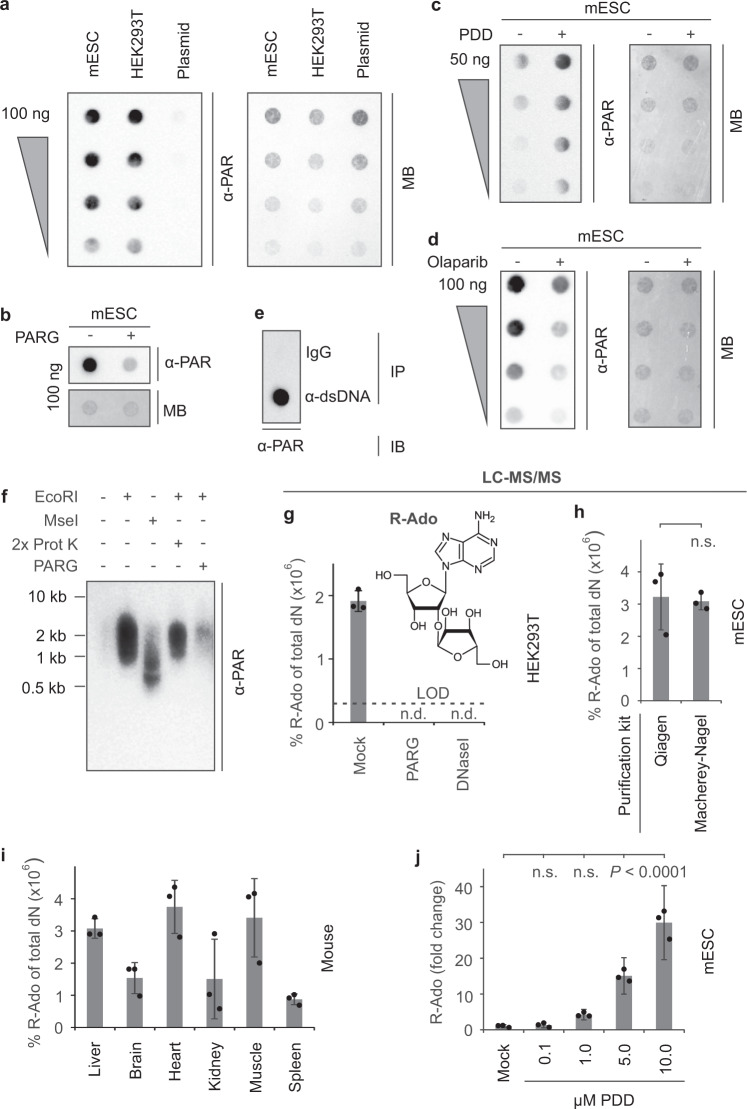
Mouse and human genomic DNA is PARylated. Dot blot analysis for PARylation of mESC, HEK293T and plasmid DNA (**a**), mESC DNA treated with PARG (**b**), DNA purified from mESCs treated with the PARG inhibitor PDD00017273 (**c**), DNA purified from mESCs treated with the PARP inhibitor olaparib (**d**), genomic mESC DNA after IP with IgG or anti-dsDNA antibody (**e**). gDNA was serially diluted (2×) in **a** and **c**, **d**; MB, methylene blue. **f** Southwestern blot analysis for PARylation of mESC DNA treated with EcoRI, MseI, Proteinase K, and PARG as indicated. Length of marker DNA is shown on the left. Southwestern is representative of three independent experiments with similar outcomes. **g** LC-MS/MS quantification of ribosyl-adenosine (R-Ado) on HEK293T DNA treated as indicated. Samples were repurified after enzyme treatments by a second column-based DNA purification to remove any PAR and DNA monomers. Dashed line, limit of detection (LOD). Data are presented as mean values ± s.d. of three biological replicates; n.d., not detected. **h–j** LC-MS/MS quantification of R-Ado (**h**) of mESC DNA isolated by two different DNA preparation kits (Qiagen Blood & Cell culture DNA kit or Macherey-Nagel NucleoBond HMW DNA kit; data are presented as mean values ± s.d. of three biological replicates; n.s., not significant by two-sided, unpaired *t* test for unequal variances), **i** of DNA from the indicated adult female mouse tissues (data are presented as mean values ± s.d. of three individual mice), **j** of DNA from mESCs treated with increasing amounts of the PARG inhibitor PDD00017273. R-Ado levels from mock-treatment is arbitrarily set to 1 (data are presented as mean values ± s.d. of three biological replicates; indicated *p* values and not significant (n.s.) as by two-sided Dunnett’s test). Source data are provided as a Source Data file.

We probed PARylation of DNA by dot blot following extensive treatment with Proteinase K to eliminate any residual proteins. We employed a widely used anti-poly ADP-ribose antibody that recognizes linear PAR chains and is free of DNA/RNA cross-reactivity (clone 10H^15^). Total DNA from mouse embryonic stem cell (mESC) and human HEK293T was positive for PAR, while plasmid DNA from *E. coli* (control) was negative (Fig. 1a). We carefully validated the specificity of the antibody signal: i) An anti-pan-ADP-ribose binding reagent (MABE1016) confirmed PARylation of mammalian DNA (Supplementary Fig. 1a) and was used hereafter in parallel with 10H anti-PAR antibody; (ii) Treatment with PAR glycohydrolase (PARG), the primary enzyme responsible for degrading cellular ADP-ribose moieties^2^, eliminated the PAR signal of auto-PARylated PARP1 protein (Supplementary Fig. 1b, c) and of mESC DNA (Fig. 1b, Supplementary Fig. 1d), outruling DNA cross-reactivity; iii) DNA PARylation was strongly enhanced when mESCs were grown in presence of PDD, a cell-permeable inhibitor of PARG^16^ (Fig. 1c, Supplementary Fig. 1e), and reduced in mESCs treated with the PARP inhibitor olaparib^17^ (Fig. 1d, Supplementary Fig. 1f), supporting anti-PAR specificity; iv) DNase I digestion erased the PAR signal, indicating that it did not stem from residual proteins or co-purifying free PAR chains released by Proteinase K digestion (Supplementary Fig. 1g). On the other hand, DNase I treatment did not affect the PAR signal of free PAR chains, ruling out that DNase I may degrade PAR chains directly (Supplementary Fig. 1g); v) Combined RNase A, RNase H, and RNase III treatment had no effect on the PAR signal from genomic DNA (Supplementary Fig. 1h), ruling out PARylation of contaminating RNA^18^; vi) Immunoprecipitation of mESC DNA using an anti-dsDNA antibody enriched for PAR chains (Fig. 1e); vii) Southwestern blot analysis of mESC DNA with anti-PAR antibody (Fig. 1f) and HEK293T DNA with both anti-PAR antibody and anti-pan-ADPr reagent (Supplementary Fig. 1i, j) yielded a signal in form of a smear only after restriction digest, indicating that PAR chains are covalently attached to high molecular weight DNA, instead of free or protein-bound PAR chains co-purifying during DNA preparation. The average molecular weight of the PAR signal smear was expectedly higher for the 6-base cutter EcoRI than for the 4-base cutter MseI. Furthermore, the PAR signal was strongly reduced by treatment with PARG but not by a second Proteinase K digest/phenol-chloroform purification (Fig. 1f); viii) Dot blot analysis of DNA from diverse human organs revealed PARylation in all tested tissues but with notable differences (Supplementary Fig. 1k), suggesting biological specificity.

To confirm mammalian DNA-PARylation antibody-independently, we employed stable isotope dilution mass spectrometry (LC-MS/MS), the gold standard in quantification of modified nucleosides. DNA from HEK293T was enzymatically digested to nucleosides and analyzed by LC-MS/MS for ribosyl-adenosine (R-Ado), a product diagnostic for linear PAR chains^19^. R-Ado analysis was highly sensitive and accurate, with a limit of detection (LOD) of 3 × 10^−17^ moles, or ~6 R-Ado molecules per mammalian genome. In DNA from HEK293T cells, R-Ado was detectable at very low levels of 2 × 10^−6^ % of total dN, corresponding to ~100 R-Ado monomers per genome (Fig. 1g). Again, PARG or DNaseI treatment reduced R-Ado signals below detection limit. R-Ado was also detected in DNA from mESCs and mouse organs up to 4 × 10^−6^ % of total dN (Fig. 1h, i). Given that PAR chains can have a chain length in the order of ~100 residues^20^, this yields an average of 1–2 PARylation sites per genome. Notably, treating mESCs with increasing doses of the PARG inhibitor PDD increased DNA PARylation up to 30-fold, indicating reversibility and high turnover of the modification (Fig. 1j).

We conclude that PARylation is a rare and reversible DNA modification occurring in primary mammalian tissues and cell lines.

### DNA PARylation requires PARP1 and is unrelated to DNA breaks

**Fig. 2:**
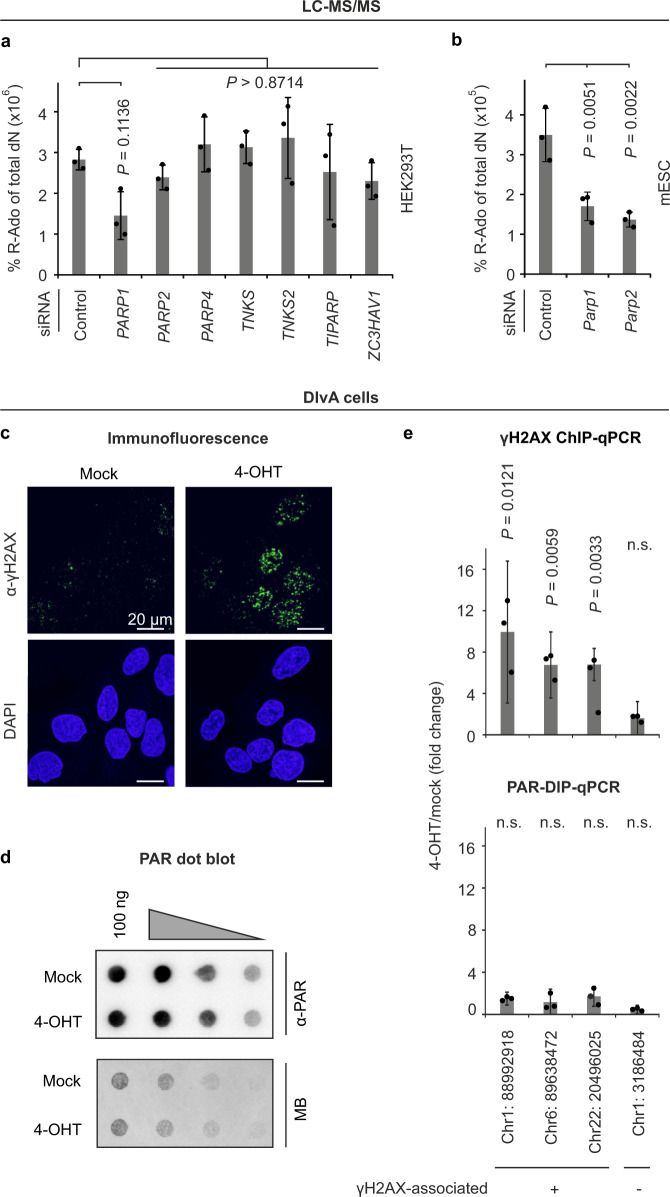
DNA PARylation requires PARP1 but is not triggered by strand breaks in vivo. LC-MS/MS quantification of ribosyladenosine (R-Ado) in 293T (**a**) and mESC (**b**) DNA after siRNA depletion of selected *PARPs*. Data are presented as mean values ± s.d. of three biological replicates; indicated *p* values as by two-sided Dunnett’s test. **c** γH2AX immunofluorescence of DIvA cells in which AsiSI nuclease expression was induced by 4-OHT treatment. DNA is stained with DAPI. Immunofluorescence is representative of three independent experiments with similar outcomes. **d** Dot blot analysis for PARylation of DNA from AsiSI nuclease expressing DIvA cells treated as in **c** with serially diluted (2×) DNA. **e** Top: γH2AX ChIP-qPCR analysis of selected genomic loci (shown at the bottom) of DIvA cells previously described to be associated with (+) or without (−) γH2AX upon 4-OHT treatment to induce AsiSI nuclease^24^. Bottom: PAR-DIP-qPCR analysis of the same loci as used for top panel. Target regions are named by chromosomal location of the AsiSI recognition site in the human hg38 reference assembly. Data are presented as mean values ± s.d. of three biological replicates; indicated *p* values and not significant (n.s.) over mock as by two-sided, unpaired *t* test for unequal variances. Source data are provided as a Source Data file.

Which enzyme might PARylate DNA in mammals? PARylation is catalyzed by the PARP family that consists of 17 and 16 members in human and mouse, respectively^21^. Based on their expression level in mESCs and HEK293T cells, we selected PARP1, PARP2, PARP4, TNKS (PARP5A) TNKS2 (PARP5B), PARP7, and ZC3HAV1 (PARP13) as candidates for further analysis (Supplementary Fig. 2a, b). In HEK293T cells, we observed a tendency for siRNA-mediated knockdown of *PARP1* but not the other tested *PARPs* to decrease global DNA PARylation while in mESCs both *Parp1* and *Parp2* knockdowns significantly decreased DNA PARylation measured by LC-MS/MS quantification of R-Ado (Fig. 2a, b).

PARP1 plays a key role in the DNA damage response, where it recognizes DNA strand breaks and PARylates nearby proteins to initiate repair^22^. In addition, PARP proteins can PARylate DNA ends in vitro and it was proposed that this may reflect a physiological role during break repair^23^. Hence, we examined whether DNA PARylation may generally localize to DNA strand breaks (DSB). We made use of DIvA cells as experimental cell system, which harbor the DNA rare cutter AsiSI that in presence of 4-hydroxytamoxifen (4-OHT) creates DSBs at defined positions across the genome and in various chromatin contexts^24^. While 4-OHT treatment of DIvA cells robustly induced gH2AX nuclear foci (Fig. 2c), there was no increase in global DNA PARylation (Fig. 2d). To specifically monitor local changes of DNA PARylation, we performed PARylated DNA immunoprecipitation (PAR-DIP)qPCR for three known AsiSI-mediated DNA break loci^24^. While 4-OHT-induced γH2AX recruitment to these loci, PAR signals were low and remained unchanged by 4-OHT (Fig. 2e). We conclude that DNA PARylation is not a general feature of DNA breaks.

### DNA PARylation by PARP1 occurs at adenosine residues in single-stranded DNA

**Fig. 3:**
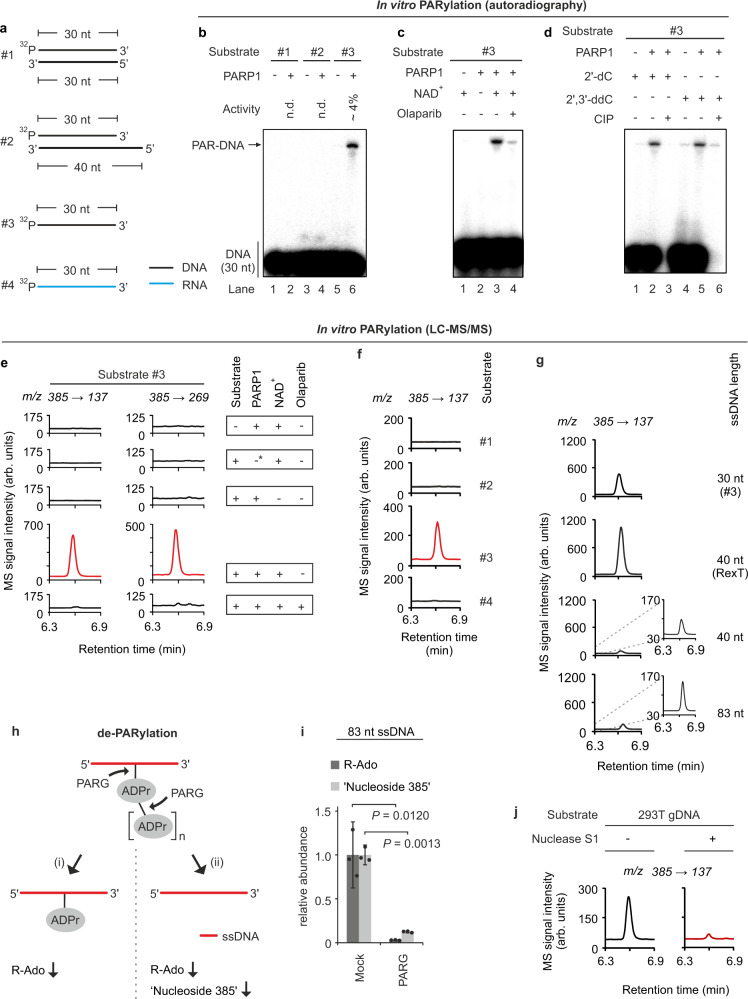
PARP1 PARylates ssDNA at a nucleobase. **a** Scheme of substrates #1–#4 used for in vitro PARylation assays. The 30 nt black strand is designated as standard oligo in the main text. ^32^P, 5’-phosphate with ^32^P-radiolabel. **b**–**d**, Autoradiography of denaturing PAGE of reaction products from in vitro PARylation assay with substrates shown in **a** in presence or absence of human PARP1, NAD^+^ and the PARP1 inhibitor olaparib. In **d**, substrate #3 contained either a 2′-dC or a 2′,3′-ddC terminal nucleotide and was treated with or without calf intestine phosphatase (CIP) post reaction. The autoradiographs are representative of three independent experiments with similar outcomes. **e**–**g** LC-MS/MS chromatograms of reaction products from in vitro PARylation assays in presence of native or heat-denatured PARP1: **e** with or without substrate #3, NAD^+^ and olaparib as indicated. Left and right panels show mass transitions corresponding to mass shifts expected for loss of a deoxyribose + ribose (*m/z* 385 → 137) and loss of a single deoxyribose (*m/z* 385 → 269) from the parental ‘nucleoside 385’. -*, heat-denatured PARP1. **f** with substrates #1–#4 as shown in **a**. Mass transition is shown as expected for loss of a deoxyribose + ribose (*m/z* 385 → 137) from the parental molecule ‘nucleoside 385’. **g** with standard substrate #3, and three additional ssDNA oligonucleotides (RexT^13^, 40 nt, 83 nt). The signal of the lower two chromatograms is magnified on the right. **h** Scheme for two alternative outcomes of de-PARylation of PARylated DNA with PARG. (i) PARG hydrolyzes the ADP ribose polymer but not the terminal ADP ribose unit at the DNA base acceptor side. LC-MS/MS analysis of the reaction products would yield decreased R-Ado and unchanged ‘nucleoside 385’ levels. (ii) In addition to polymer degradation, PARG cleaves the linkage of the ADP ribose and the DNA base acceptor side resulting in both decreased R-Ado and ‘nucleoside 385’ levels. **i** LC-MS/MS quantification of R-Ado and ‘nucleoside 385’ on an 83mer ssDNA oligo after in vitro PARylation by PARP1 followed by mock or PARG treatment of the reaction products as indicated. Data are presented as mean values ± s.d. of three independent experiments; indicated *p* values as by two-sided, unpaired *t* test for unequal variances. **j** PARP1 forms ‘nucleoside 385’ with single stranded genomic DNA. LC-MS/MS chromatograms for ‘nucleoside 385’ of reaction products from in vitro PARylation. Genomic DNA from 293T cells pretreated or not with Nuclease S1 was incubated with PARP1 and NAD^+^. Source data are provided as a Source Data file.

DNA-PARylation, if uncontrolled, may be deleterious to cells, so there must be mechanisms that provide specificity. PARP1 may display substrate specificity towards certain types of DNA. To analyze substrate specificity, we performed in vitro PARylation reactions with PARP1 (Supplementary Fig. 3a) and an arbitrary 30 nt DNA-oligonucleotide (hereafter referred as “standard oligo”) carrying a ^32^P-end-labeled 5′-phosphate. We tested the standard oligo as ssDNA and dsDNA with either blunt- or 3′-recessed end (Fig. 3a). Reaction products were analyzed by denaturing gel electrophoresis, where PARylated DNA accumulates at the top of the gel^13^. Interestingly, ssDNA was robustly PARylated, unlike dsDNA (Fig. 3b). The reaction required the PARP1 co-substrate NAD^+^ and was inhibited by the PARP-inhibitor olaparib (Fig. 3c). DNA PARyation was not due to spontaneous- or PARP1-catalyzed addition of preexisting PAR chains to the standard oligo (Supplementary Fig. 3b) and PARP1 did not PARylate ssRNA (Supplementary Fig. 3c, d).

Since PARP1 was reported to PARylate both 5′- and 3′-terminal phosphates of complex DSB-mimicking oligonucleotides in vitro^10,13^, we released terminal ^32^P-end-labeled 5′-phosphate with calf intestine phosphatase (CIP). If PARylation occurs on the labeled 5′-phosphate, the modification should protect the label against CIP treatment. However, CIP treatment erased the signal of both the unmodified and PARylated standard oligo, thus excluding PARylation of the DNA 5′-phosphate terminus (Fig. 3d). Conversely, the 3′-end was also unmodified, since PARylation occurred with an oligo terminating in a dideoxy-cytosine (2′3′-ddC), i.e., lacking a 3′-OH acceptor site. As control, the PAR signal still decreased upon CIP treatment, ruling out alternating PAR-acceptor sites (Fig. 3d). These results suggest that in ssDNA, PARylation occurs at DNA bases rather than at termini. We also tested an oligo (RexT) previously shown to be 5′-terminal PARylated by PARP1^13^. Following in vitro PARylation, CIP treatment removed ~70% of the signal, confirming that while there is 5′-phosphate modification, PARylation mostly occurs internally (Supplementary Fig. 3e).

To identify the PAR acceptor site on ssDNA, we adopted a method^19^ that enzymatically degrades the PAR chains and leaves a single ribose remnant on the PAR acceptor molecule. Conveniently, this procedure also degrades DNA to single nucleosides. Consequently, we degraded the in vitro PARylated standard oligo and screened the products by LC-MS/MS for ribosylated nucleosides, i.e., containing both a ribose moiety remaining from PARylation and a deoxyribose. Intriguingly, we identified a positively charged ion with a mass over charge ratio (*m/z*) of 385 (‘nucleoside 385’) and mass transitions that fit to loss of a ribose and a deoxyribose (*m/z* = 385 → 137), as well as loss of a single deoxyribose (*m/z* = 385 → 269), supporting the presence of both sugars on the nucleobase (Fig. 3e). Generation of ‘nucleoside 385’ was dependent on active PARP1, the standard oligo, NAD^+^, and was sensitive to olaparib treatment. We observed no signals for ‘nucleoside 385’ from dsDNA or ssRNA after PARP1 incubation (Fig. 3f). Thus, levels of ‘nucleoside 385’ closely correlate with levels of radiolabeled PARylation products (Fig. 3b, c, Supplementary Fig. 3c). 'Nucleoside 385' was also formed with varying efficiency using three additional ssDNA oligos of different sequences and lengths, including RexT^13^ (Fig. 3g). PARG treatment of a PARylated 83mer ssDNA strongly reduced the levels of ‘nucleoside 385’, indicative of reversibility of base PARylation in vitro (Fig. 3h, i). Furthermore, we detected ‘nucleoside 385’ when using HEK293T genomic DNA instead of synthetic DNA oligonucleotides as substrate for PARP1 in vitro PARylation. Again, the reaction was ssDNA-dependent as pre-incubation of gDNA with Nuclease S1 prevented ‘nucleoside 385’ formation (Fig. 3j).

**Fig. 4:**
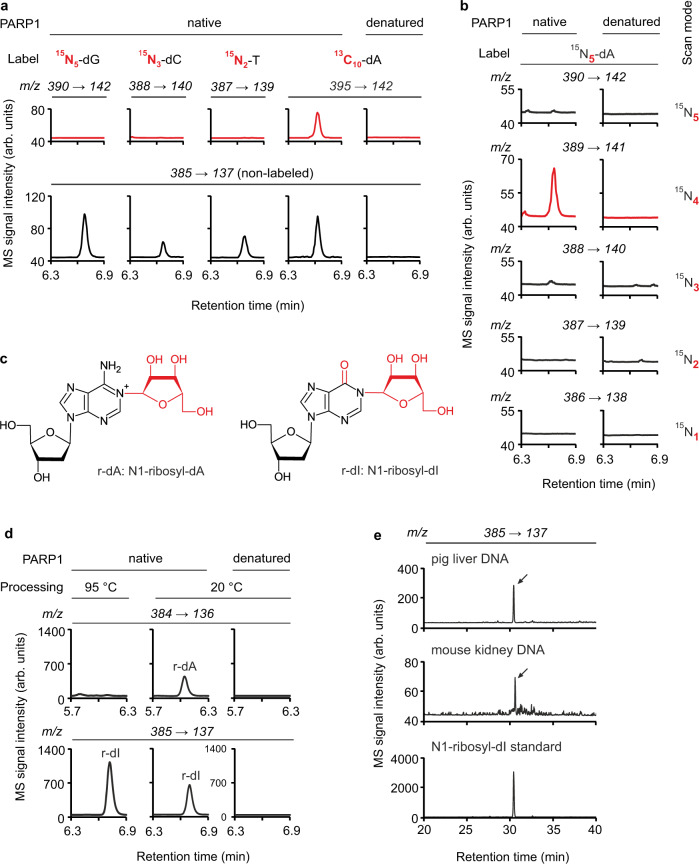
DNA PARylation occurs at the N1-position of adenosine residues in vitro and in vivo. **a** LC-MS/MS chromatograms of reaction products from in vitro PARylation assays in presence of native or heat-denatured PARP1 with four 83mer ssDNA substrates each bearing one type of heavy isotope-labeled nucleoside as indicated. Top, scan for signals with *m/z* transitions expected for a mass shift of ‘nucleoside 385’ from each label. Bottom, detection of signals that correspond to non-labeled ‘nucleoside 385’. Note, all four substrates contain a mixture of the respective labeled and unlabeled nucleoside. **b** LC-MS/MS chromatograms of reaction products from in vitro PARylation assays as in **a** but with a ^15^N_5_-dA-labeled 83mer ssDNA substrate. Reaction products were scanned for signals with *m/z* transitions that correspond to mass shifts of +5 to +1 of ‘nucleoside 385’ (from top to bottom). **c** Spontaneous deamination of N1-ribosyl dA (r-dA, attached ribose moiety in red) leads to N1-ribosyl-dI (r-dI, ribose moiety and O^6^ in red). **d** LC-MS/MS chromatograms of reaction products from in vitro PARylation assays as in **a** but with substrate #3 in which reaction products were either processed at 95 °C or 20 °C before mass spec analysis, and screened for signals with *m/z* transitions expected for N1-ribosyl-dA (r-dA, *m/z* 384 → 136, top) or N1-ribosyl-dI (r-dI, *m/z* 385 → 137, bottom). **e** LC-MS/MS chromatograms of N1-ribosyl-dI, (arrows, *m/z* 385 → 137) enriched from 5 mg pig liver and 2.5 mg mouse kidney DNA and compared to the reaction product of an in vitro PARylation assay with PARP1 on substrate #3.

To identify the ribosylated base, we first used ssDNA oligos homopolymeric for all four canonical nucleosides but could not detect ‘nucleoside 385’ after in vitro PARylation (Supplementary Fig. 4a). The result suggests a requirement of base PARylation for certain sequence contexts and/or secondary structures in ssDNA. Next, we in vitro-PARylated four 83mer ssDNA substrates with PARP1, each containing one isotopically labeled nucleotide (^15^N_5_-dG, ^15^N_3_-dC, ^15^N_2_-T or ^13^C_10_-dA, respectively), and scanned the reaction products for the mass of a respective heavy ‘nucleoside 385’. The only reaction that yielded the expected mass shift of ‘nucleoside 385’ was the ^13^C_10_-dA 83mer oligo, indicating adenine as the PAR acceptor base (Fig. 4a). Interestingly, replacing carbon-labeled ^13^C_10_-dA by nitrogen-labeled ^15^N_5_-dA on the 83mer substrate, we could not detect the expected ‘nucleoside 385’ + 5 Da = 390 ion product but instead a ‘nucleoside 385’ + 4 Da = 389 ion product (Fig. 4b). The result indicates that the ‘nucleoside 385’ adenosine derivative lost one labeled nitrogen atom before, during, or after PARylation. Indeed, adenosine can lose a nitrogen by deamination to inosine^25^. Strikingly, the predicted *m/z* for ionized N1-ribosyl-deoxyinosine (M_r_ + H^+^) matches exactly the observed *m/z* of ‘nucleoside 385’. Hence, we first reasoned that inosine formed by deamination of adenosine might be the true acceptor residue for PARylation. We therefore in vitro PARylated the standard 30 nt oligo, in which all six dA residues were substituted by dI (Supplementary Fig. 4b). However, we detected neither significant ‘nucleoside 385’ by LC-MS/MS nor any PARylated oligo in radioactive PARylation assay (Supplementary Fig. 4c, d) excluding inosine as acceptor for PARylation. We next considered that deamination might not be cause but consequence of PARylation of dA, i.e., ribosylated dA might be prone to deamination. While deamination of adenosine is slow in intact DNA^26^, N1-substituted dA adducts readily deaminate^27,28^. Hence, we hypothesized that N1-ribosyl-dA is the precursor of N1-ribosyl-dI (Supplementary Fig. 4e). However, the deaminase inhibitor pentostatin added to the PARylation reaction as well as to the enzymatic degradation step had no effect on the levels of ‘nucleoside 385’ or the occurrence of a signal expected for N1-ribosyl-dA (Supplementary Fig. 4f). This result argues against enzymatic deamination by contaminating deaminases and points towards spontaneous deamination. To reduce spontaneous deamination, we omitted a 95 °C denaturation step in the DNA processing protocol and avoided prolonged sample storage prior to LC-MS/MS analysis. Using these precautions, we indeed detected in the PAR reaction of the standard oligo besides ‘nucleoside 385’ a new signal with an *m/z* matching N1-ribosyl-dA (*m/z* = 384 → 136) (Fig. 4c, d). Moreover, when we analyzed under these conditions a ^15^N_5_-dA-labeled 83mer DNA PARylation product, we detected two signals with the same *m/z* transition but different LC-retention times, as expected for ^15^N_5_-labeled N1-ribosyl-dA and ^15^N_4_-labeled N1-ribosyl-dI (Supplementary Fig. 4g, h). Further analysis revealed that deamination is slow on PARylated-dA in intact DNA but accelerated on nucleosides (Supplementary Fig. 5a–c). We conclude that PARP1 catalyzes PARylation of ssDNA on adenosine-N1 forming N1-Poly(ADP-ribosyl)-dA.

To demonstrate that DNA PARylation occurs on N1-adenosine in vivo, we developed an LC-MS/MS protocol to detect in hydrolyzed gDNA the diagnostic deamination product, N1-ribosyl-dI. Expecting it to be ultra-rare, we used several milligrams of pig liver and mouse kidney DNA. Separating ssDNA from dsDNA and enriching by HPLC, we indeed detected N1-ribosyl-dI in DNA from both tissues (Fig. 4e).

## Discussion

PARylation is an important posttranslational modification of proteins that is involved in numerous processes. In mammalian cells, PARylation is thought to be limited to proteins. PARylation of mammalian DNA therefore comes as a surprise. While rare in abundance, DNA PARylation is widespread in mammalian DNA from different cell lines, tissues, and species. We provide evidence that DNA PARylation by PARP1 occurs on adenosine N1, which is indeed the most nucleophilic atom of adenine^29^, providing a rationale for its PARylation. We also show that PARP1 specifically acts on ssDNA, likely due to greater accessibility of unpaired adenosine residues, because the adenine N1 position is engaged in dsDNA base-pairing. Certain bacterial, insect and mollusc mono-ADP-ribosyltransferases are also known to modify ssDNA and dsDNA on thymidine and on the N2 position of guanosine^4–6,8,9^, but modification of adenosine has, to our knowledge, not been described.

We show that PARylation requires PARP1 and PARP2, two major PARP enzymes that have been widely studied in protein PARylation^3^. Indeed, PARP1 is known to recognize distortions in the DNA helical backbone and its enzymatic activity is activated by hairpins, cruciforms, and stably unpaired regions in double-stranded DNA^30^. Moreover, PARP1 is involved in various processes that also involve ssDNA, e.g., replication, transcription, or DNA damage repair^22^. However, we show that DNA PARylation is likely not a generalized feature of DNA breaks. Results from in vitro DNA PARylation by PARP1 support that sequence contexts or secondary structure of ssDNA are essential. Furthermore, given the low efficiency of DNA PARylation we observe in vitro, PARP1 in vivo may also employ cofactors^31,32^ that enhance the reaction and provide additional specificity.

PARylation of proteins is reversible by PAR-glycohydrolases^33^ and we provide evidence that DNA PARylation is also reversible in vitro and in vivo by PARG, indicating that it is dynamic and subject to regulation. However, since the PARG inhibitor PDD that we employed is not selective for PARG^34^, other glycohydrolases may also reverse DNA PARylation in vivo, including TARG1, MACROD1/2, and ARH3^33^.

A key open question that remains to be addressed is where in the genome PARylation occurs. The prediction of our in vitro PARylation assays is that the PARP1 target is single stranded DNA, which physiologically occurs in different contexts, for example in DNA:RNA hybrids (R-loops), replicating DNA, DNA repair intermediates, G-quadruplex DNA, cDNA, and DNA tandem repeats. To map DNA PARylation genome-wide, we attempted PARylated DNA immunoprecipitation followed by next generation sequencing (PAR-DIP-seq). However, standard sample workup during next generation sequencing precludes amplification and detection of the PARylated DNA strand because of steric hindrance by PAR chains that stall DNA polymerases. Thus, methods need to be developed that overcome this technical challenge in the future. PAR-DIP-seq will then allow addressing the physiological substrate specificity and the biological relevance of this mammalian DNA modification.

Another challenge in addressing the biological function of PARylation by PARP1 is its pleiotropy, the vast number of substrates and processes in which this protein is involved, including transcription and DNA repair^22^, which render it difficult to interpret loss-of-function effects and establish causality. Hence, a separation-of-function PARP1 mutant that is deficient in PARylation of DNA but not of protein will be an ideal tool to address the physiological role of DNA vs. protein PARylation in the future.

Our study establishes the conceptual and methodological framework to address these and other emerging questions.

## Methods

### Cell culture and transfection

mESC clone WT #4^35^ was cultured on tissue culture plates coated with 0.1% Gelatin (Millipore) in 2i medium (Neurobasal—DMEM/F-12 medium (Gibco), supplemented with 1x N2 (Gibco), 1x B27 (Gibco), 2 mM L-Glutamine (Gibco), 1000 U/ml Leukemia inhibitory factor (LIF, Millipore), 100 U/ml PEN-STREP (Gibco), 1 µM PD0325901 (Sigma), 3 µM CHIR99021 (Sigma), 50 µg/ml BSA) at 37 °C in 5% CO_2_ and 20% O_2_. HEK293T (ATCC number CRL-11268) and DIvA cells^24^ (kind gift from G. Legube) were cultured in DMEM (Gibco) supplemented with 10% FBS Gold (PAA), 2 mM L-glutamine, and 100 U/ml PEN-STREP at 37 °C in 5% CO_2_ and 20% O_2_. All cell lines were tested negative for mycoplasma contamination.

Cells were transiently transfected with siRNAs with Lipofectamine 2000 (Life Technologies) according to the manufacturer’s instructions. siRNA knockdown efficiencies were routinely assessed by reverse transcription (RT)-coupled qPCR, and were at least 60% efficient. For PARG inhibition, mESCs were treated with 0.1–10 µM PDD00017273 (Sigma) for 48 h. PARP inhibititon was for 24 h with 1 µM olaparib (Selleckchem, S1060).

### Protein purification

#### PARG

Plasmid pGEX6P3-hPARGfl^36^ encoding a codon-optimized full-length human poly (ADP-ribose) glycohydrolase (PARG) was a gift from J.C. Amé. Expression and purification of PARG was essentially as described before^36^ using the *E. coli* expression strain BL21-CodonPlus(DE3)-RIL (Stratagene). Purified PARG was tested for enzymatic activity on auto-PARylated PARP1. For auto-PARylation, 2 µg PARP1 (Enzo Life Sciences) was incubated in 10 µl of 50 mM Tris-HCl pH 8.0, 100 mM NaCl, and 10 µM NAD^+^ for 30 min at 37 °C. To test for PAR glycohydrolase activity, 2 µg PARG was incubated with 0.5 µg auto-PARylated PARP1 in 20 µl 1x PARG buffer (50 mM Tris pH 8.0, 2 mM MgCl_2_, 1 mM DTT, 100 mM NaCl) at 30 °C overnight. The reaction products were analyzed for PARylation by dot blot (see below).

#### PARP1

Human full-length *PARP1* cDNA (pCMV3-HA-PARP1, SinoBiological) was inserted into a pFastBac-vector, encoding an N-terminal His_6_-MBP-HRV-3C-tagged fusion protein. His_6_-MBP-HRV-3C-PARP1 was produced in 1.2 l SF9 insect cells using the Baculovirus expression system. PARP1 was purified essentially as described^37^. The His-MBP-tag was removed using 3 C protease over night at 4 °C prior to the Heparin chromatography step, and the final purified protein was stored in 25 mM HEPES-KOH pH 7.4, 300 mM NaCl, 1 mM EDTA, 1 mM DTT, 10% glycerol.

### Nuclease and PARG treatments

PARG treatment of genomic DNA was done with 0.5 µg purified PARG per 1 µg of DNA in 1x PARG buffer (50 mM Tris-HCl pH 8.0, 2 mM MgCl_2_, 1 mM DTT, 100 mM NaCl) at 30 °C overnight, followed by Proteinase K (Qiagen, 10 µg per 1 µg DNA, 4 h, 50 °C) digestion and phenol/chloroform extraction of DNA. DNase I digestion was performed with 1 U DNase I (ThermoFisher) per 1 µg of DNA or PAR polymer (R&D Systems) for 1 h at 37 °C according to manufacturer’s instruction, and followed by phenol/chloroform extraction. For triple-RNase treatment, genomic DNA/PAR polymer was consecutively incubated with 10 µg RNase A (ThermoFisher) per 1 µg of DNA/PAR polymer in TE buffer supplemented with 300 mM NaCl, 4 U ShortCut RNase III (NEB) per 1 µg of DNA/PAR polymer, and 2.5 U RNase H (NEB) per 1 µg DNA/PAR polymer according to supplier’s instructions for 1 h at 37 °C. DNA/PAR polymer was phenol/chloroform extracted after each single reaction. Nuclease S1 treatment was done with 1 U Nuclease S1, respectively, per 1 µg genomic DNA for 1 h at 37 °C, and with phenol/chloroform extraction after the reaction. For Southwestern analysis, DNA was treated with 20 U EcoRI (NEB) or 20 U MseI (NEB) per 1 µg of DNA according to manufacturer’s instructions for 1 h at 37 °C and subsequent phenol/chloroform extraction.

### Dot blot

Genomic DNA was isolated using the Blood & Cell Culture Kit (Qiagen) according to manufacturer’s instruction, and with overnight Proteinase K treatment, dissolving of DNA in presence of 20 µg/ml RNase A and a final phenol/chloroform extraction. Genomic DNA from human adult normal tissues was purchased from Amsbio (brain, HG-201; thymus, HG-702; heart, HG-801; skeletal muscle, HG-102; liver, HG-314; pancreas, HG-313; kidney, HG-901; spleen, HG-701; testis, HG-401; placenta, HG-413). Human tissue DNA was treated overnight with Proteinase K (0.3 mg/ml final concentration) followed by incubation with RNaseA (20 µg/ml final concentration) and phenol/chloroform extraction. Plasmid DNA (pEGFP-C1, Clontech) was prepared by QIAprep Spin Miniprep Kit. DNA was denatured for 10 min at 95 °C in 500 µl 2x SSC and loaded on a 6x SSC equilibrated Hybond®-N + Blotting Membrane using a Bio-Dot microfiltration unit (Bio-Rad) according to manufacturer’s instructions. DNA was crosslinked to the membrane with a Stratalinker UV Crosslinker using the auto crosslink mode. DNA loading on membrane was visualized with 0.1% methylene blue in 0.5 M sodium acetate. For immunodetection of DNA PARylation, membranes were washed twice in TBST, blocked with 5% non-fat dry milk, incubated overnight with a 1:5000 dilution of an anti-poly (ADP-ribose) mouse monoclonal antibody (Trevigen, 4335-MC-100, clone 10HA) or a 1:5000 dilution of anti-pan-ADP-ribose biding reagent (Merck, MABE1016), washed 3x with TBST, incubated for 1 h at room temperature with a 1:5000 dilution of a goat anti-mouse IgG-HRP conjugate (Dianova, 115-035-146) or a goat anti-rabbit IgG-HRP conjugate (Dianova, 111-035-144) and washed 3x with TBST. Chemiluminescence was induced with SuperSignal West Femto ECL solution (ThermoFisher) according to manufacturer’s instructions and recorded on a Bio-Rad ChemiDoc imaging system using the Image Lab Software v. 6.1.0.

### dsDNA immunoprecipitation

Genomic DNA of mESC was prepared with Blood & Cell Culture Kit (Qiagen) as described above. DNA immunoprecipitation was performed as described below using 5 µg mouse anti-ds DNA antibody (Abcam, ab27156, clone 35I9 DNA) or 5 µg mouse IgG control (Sigma, I8765). After final resuspension, 5 µl of each sample was subjected to dot blot analysis as described above.

### Southwestern blotting assay

Genomic DNA was prepared with Blood & Cell Culture Kit (Qiagen) as described above and separated by electrophoresis on a 1% agarose gel (1 µg DNA per sample) followed by capillary blotting to a Hybond-N + Membrane (GE Healthcare) using 20x SSC as blotting buffer. Crosslinking of DNA and immune-detection of DNA PARylation was as described above.

### Quantification of ribosyl-adenosine (R-Ado) by LC-MS/MS

#### Generation of ^15^N-labeled ribosyl-adenosine

Stable isotope labeling of PAR polymers was performed by two consecutive enzymatic reactions essentially as described^19^. Briefly, ^15^N_5_-NAD^+^ was generated in a reaction containing 2 mM ^15^N_5_-labeled ATP (Silantes), 2 mM β-nicotinamide mononucleotide (N3501, Sigma) and 0.1 µg/µl nicotinamide-nucleotide adenylyltransferase (ProSpec, ENZ-1002) in 25 mM Tris-HCl, pH 7.5, 20 mM MgCl_2_ for 1 h at 37 °C. Isotopically labeled NAD^+^ was used for PARP1 auto-PARylation as described above followed by phenol/chloroform extraction of the labeled PAR polymers. Purified ^15^N-PAR chains were degraded with nuclease P1 (NP1, Roche), snake venom phosphodiesterase (SVP, Worthington) and fast alkaline phosphatase (FastAP, Fermentas). The resulting ^15^N_5_-ribosyl-adenosine (^15^N_5_-R-Ado) and ^15^N_5_-diribosyl-adenosine (^15^N_5_−2R-Ado) were separated on an Agilent 1290 Infinity Binary LC system (Agilent Technologies) using a ReproSil 100 C18 column (Jasco). Isotopically labeled R-Ado and 2R-Ado were identified by analytical HPLC in tandem with triple quadruple mass spectrometry (Agilent 6490, Agilent Technologies) and purified by preparative HPLC. Concentrations of labeled R-Ado and 2R-Ado were experimentally determined by LC-MS/MS with defined concentrations of unlabeled reference R-Ado and 2R-Ado obtained from degraded PAR polymers (R&D systems, molar extinction coefficient 13.5 mM^−1^ cm^−1^, assumed average length of 150 monomers).

#### Genomic DNA preparation and LC-MS/MS analysis

Genomic DNA was prepared with Blood & Cell Culture Kit (Qiagen) as described above but without a final phenol/chloroform extraction. PARG- and DNaseI-treated DNA was subjected to a second column purification. Mouse organ tissues were obtained from 7 to 8 weeks old female C57BL/6 J mice (Translational Animal Research Center, Mainz). Tissues were homogenized with an Ultra-TURRAX disperser (IKA) prior to DNA preparation. About 10 µg of DNA was degraded to nucleosides with NP1 (Roche), SVP (Worthington) and FastAP (Fermentas). An equal volume of isotopic standard mixture ^15^N_5_-dG (Silantes), ^15^N_5_^13^C_10_-dA (Silantes) and self-synthesized ^15^N_5_-R-Ado and ^15^N_5_−2R-Ado (see above) was added to the DNA samples and ~10 µg of total DNA was injected for LC-MS/MS analysis. Quantitative analysis was performed on an Agilent 1290 Infinity Binary LC system (Agilent Technologies) using ZORBAX SB-C18 column (Agilent Technologies, 5 mm, 2.1 × 50 mm) coupled to an Agilent 6490 triple quadrupole mass spectrometer. Quantification of R-Ado and R2-Ado by LC-MS/MS was performed according to the published protocol^19^ with specific changes: Elution was performed with 5 mM ammonium acetate pH 6.9 and acetonitrile (ACN), the flow was first linearly increased from 0.3 ml/min to 0.38 ml/min in 0–10.5 min, then switched to 0.5 ml/min for 10.5–14.5 min, and 0.3 ml/min for 14.5–15.5 min. The column was kept at 30 °C. The gradient was: 0–3 min, 0% ACN; 3–7.5 min, 0–5% ACN; 7.5–10.5 min, 5 % ACN, 10.5–12.5 min 5–50% ACN; 12.5–15.5 min, 0% ACN. The MS source-dependent parameters were as follow: gas temperature 110 °C, gas flow 19 l/min (N_2_), Nebulizer 25 psi, sheath gas heater 375 °C, sheath gas flow 11 l/min (N_2_), capillary voltage 2000 V (positive mode), nozzle voltage 0 V, fragmentor voltage 300 V, high pressure RF 150 V, and low pressure RF 60 V. Compound dependent parameters are listed in Supplementary Table 2. Note, in none of the gDNA samples we observed signals for 2R-Ado, the expected product for branched PARylation. R-Ado quantification is shown over dN as calculated from total dG and dA signals. To calculate LOD of R-Ado molecules per genome, the LOD of the method (3 × 10^−17^ moles or 1.8 × 10^7^ molecules of R-Ado) was divided by the amount of genomes in 10 µg injected DNA, i.e., ~3.2 × 10^6^ mouse or human genomes (3.1 pg average weight per genome).

### LC-MS/MS for base PARylation

#### Generation of 83mer ssDNA substrates with isotopically labeled nucleotides

Asymmetric PCR^38^ was used to generate 83mer ssDNA substrates with heavy isotope labeled nucleotides. Each PCR reaction contained 1000 nM forward primer, 50 nM reverse primer, a 83mer synthetic template DNA (primer and template sequences are listed in Supplementary Table 1) and a dNTP-mixture in which one unlabeled dNTP was substituted with the respective heavy isotope-labeled dNTP (^15^N_5_-dGTP, ^15^N_3_-dCTP, ^15^N_2_-TTP, ^13^C_10_-dATP, or ^15^N_5_-dATP, Silantes). Amplification was performed in 50 cycles. Subsequently, excess primers and dNTPs were removed by Amicon Ultra-0.5 ml centrifugal filter units (Millipore) according to manufacturer’s instructions. The concentrated ssDNA was used for in vitro ADP-ribosylation and LC-MS/MS analysis as described below. Note, the 83mer ssDNA substrates contain a mixture of the respective heavy and natural nucleotide due to usage of unlabeled primer sequences.

#### DNA preparation and LC-MS/MS analysis

Degradation of in vitro PARylated DNA and LC-MS/MS conditions were essentially as described above for R-Ado quantification except that the MS source-dependent parameters for capillary voltage were 2200 V (positive mode), and high pressure RF was 130 V. When indicated, DNA was not heat-denatured at 95 °C for 5 min prior to degradation, and 100 nM pentostatin (Sigma) was added to the degradation mixture. Ribosyl-dA or ribosyl-dI quantification is shown over total dA. Compound dependent parameters are listed in Supplementary Table 2.

#### Enrichment of ribosyl-deoxyinosine (R-dI) from gDNA

Male adult pig liver (*Sus scrofa domestica*, University Medical Center, Mainz) and kidneys dissected from 8 to 12 weeks old male C57BL/6J mice (Translational Animal Research Center, Mainz) were homogenized with a Dounce homogenizer or an Ultra-TURRAX disperser (IKA). These experiments did not require ethical approval since mouse and pig organs were obtained from collaborators.

Genomic DNA was prepared with Blood & Cell Culture Kit (Qiagen) as described above omitting the final phenol/chloroform extraction but including an additional RNase A treatment (50 µg RNase A per 300 µg gDNA at 0.3 mg/ml) in 2 mM Tris-HCl pH 7.5, 18 mM ammonium acetate for 30 min at 37 °C followed by ethanol precipitation. Degradation of ssDNA was performed with 1400 units of Nuclease S1 per 1 mg of gDNA in 5 mM ammonium acetate pH 5.6, 0.2 mM ZnCl_2_ for 60 min at 37 °C using a total amount of 5 and 2.5 mg of pig liver and mouse kidney DNA, respectively, at 0.7 mg/ml. Degraded ssDNA was separated from undigested dsDNA by an Amicon ®Ultra-0.5 ml 30 kDa filter unit (Millipore). The ssDNA collected from the flow through was hydrolyzed with 0.4 U of NP1, 2 U of SVP and 20 U of FastAP per 1 mg of starting gDNA amounts followed by enzyme removal through an Amicon ®Ultra-0.5 ml 10 kDa filter unit (Millipore). The flow through was concentrated ~20× in a Concentrator plus (Eppendorf) at 4 °C. R-dI was enriched on an Agilent 1290 Infinity Binary LC system (Agilent Technologies) using a 250 mm × 4.6 mm ReproSil 100 C18 3 μm (Jasco) by sequential runs with 0.8 mg of starting DNA amount per injection. LC was performed with 5 mM ammonium acetate pH 6.9 and ACN, the flow was at 0.5 ml/min for 0–40 min, then gradually increased to 1 ml/min for 40–50 min, gradually decreased to 0.5 ml/min for 50–55 min. The gradient was: 0–15 min, 0% ACN; 15–35 min, 0–15% ACN; 35–40 min, 15–50 % ACN, 40–45 min 50% ACN; 45−55 min, 0% CAN at 30 °C. The collection window was from 29.9-30.9 min as determined empirically from in vitro generated R-dI, which peaks at ~30.5 min. The collected fractions were pooled, concentrated to ~20 µL and analyzed in a single analytical LC-MS/MS run using the same column and LC conditions as for R-dI purification. Data were collected with software Agilent MassHunter Workstation v. B06.00 and B09.00. Analysis perfomed with the software Agilent MassHunter Quantitative analysis v. B05.02 and B09.00, and Agilent MassHunter Qualitative analysis v. B06.00 and B08.00 Compound dependent parameters are listed in Supplementary Table 2.

### Quantitative real time PCR (qPCR)

Quantitative real time PCR was performed on a LightCycler 480 (Roche) in technical duplicates using the Universal ProbeLibrary technology (Roche) in combination with the supplier’s LightCycler 480 Probes Master. Quantitative analysis was performed with LightCycler 480 software v. 1.5.1.62 (Roche). For RT-coupled qPCR, RNA was isolated using the RNeasy mini kit (Qiagen) following the manufacturer’s instructions. cDNA synthesis was performed with SuperScript II Reverse Transcriptase (ThermoFisher). Primer sequences and hydrolysis probe numbers are listed in Supplementary Table 1.

### H2A.X chromatin immunoprecipitation (ChIP)

DIvA cells were treated with 300 nM 4-OHT (Sigma, H6278) or mock treated for 24 h before harvesting. ChIP assay was carried out as described^24^ using 200 µg of sonicated chromatin and 2 µg mouse monoclonal anti-phospho-Histone H2A.X antibody (Millipore, 05-636-I, clone JBW301) per sample. ChIP efficiencies were calculated using the percent input method after qPCR and are shown as fold change over mock treatment.

### PARylated DNA immunoprecipitation (PAR-DIP)

Genomic DNA was prepared with Blood & Cell Culture Kit (Qiagen) as described above. DNA was sonicated in a Bioruptor (Diagenode) according to manufacturer’s instructions to generate fractions between 300 and 500 bp followed by phenol/chloroform extraction and ethanol precipitation. DNA immunoprecipitation was performed overnight at 4 °C with 5 µg of sonicated DNA and 5 µg of anti-poly (ADP-ribose) mouse monoclonal antibody (Trevigen, 4335-MC-100, clone 10HA) in 500 µl of 1X IP buffer (10 mM Na-phosphate pH 7.0, 140 mM NaCl, 0.05% Triton X-100). As input 1% of sonicated DNA was kept separately. Antibodies were captured by 2 h incubation with Dynabeads Protein G (ThermoFisher, 40 µl per IP sample) pre-washed with 0.1% BSA. Following a three-time wash of beads with 700 µl IP buffer, Proteinase K digestion (0.3 mg/ml final concentration) was performed on beads and input samples for 3 h at 50 °C. Finally, DNA was phenol/chloroform extracted, ethanol precipitated and resuspended in 20 µl nuclease-free H_2_O. Target regions of γH2AX-associated and -not associated AsiSI sites (Supplementary Table 1) were chosen based on a previous analysis^24^. PAR-DIP efficiencies were calculated using the percent input method after qPCR and are shown as fold change over mock treatment.

### Immunofluorescence

Diva cells grown on coverslips and treated or mock treated for 24 h with 300 nM 4-OHT were fixed in 4% formaldehyde solution, neutralized with 200 mM glycine, permeabilized in 0.2% Triton X-100 and blocked with 5% BSA. Primary antibody incubation was done overnight at 4 °C using a mouse monoclonal anti-phospho-Histone H2A.X antibody (Millipore, 05-636-I, clone JBW301, 1:300 dilution in 1% BSA) followed by secondary antibody incubation for 2 h at room temperature using a goat anti-mouse IgG Alexa Fluor 488 conjugate (ThermoFisher, A-11029, 1:500 dilution in 1% BSA). DNA was stained 10 min in 0.5 µg/ml DAPI and coverslips mounted with ProLong Gold (ThermoFisher). Images were acquired using a Leica TCS SP5 confocal microscope and the Leica Application Suite software v. 2.4.1. Images were processed with Image J v. 1.52r.

### In vitro ADP-ribosylation assay

End-labeling of DNA was performed with 200 nM oligonucleotide with [γ−32P]ATP (PerkinElmer) and T4 polynucleotide kinase (NEB) according to the manufacturer’s instructions. 100 nM labeled DNA was hybridized in SSC buffer (150 mM NaCl and 15 mM trisodium citrate) with 100 nM complementary DNA or RNA oligonucleotides (Supplementary Table 1). Unincorporated [γ−32P]-ATP was removed by G-25 Quick Spin columns (GE Healthcare). ADP-ribosylation assay was performed with 1 nM single-stranded or duplex oligonucleotides, 1 mM NAD^+^, 20 nM recombinant PARP1 and 200 nM olaparib if indicated in ADPR buffer (20 mM HEPES-KOH, pH 7.6, 50 mM KCl, 1 mM DTT, 100 μg/ml BSA) for 60 min at 37 °C followed by treatment with 5 U alkaline phosphatase (CIP, NEB) if indicated. To test for PAR chain addition, ADP-ribosylation assay was performed in presence of 0.1–10 µM of PAR polymer (Trevigen, molar extinction coefficient 13.5 mM^−1^ cm^−1^). The reaction was stopped by addition of 50 ng/µl Proteinase K for 30 min at 56 °C. Reaction products were denatured in 1x Novex TBE-Urea Sample Buffer (ThermoFisher) and analyzed on 10% Novex TBE-Urea Gels (ThermoFisher) according to manufacturer’s instruction. Phosphorimaging was performed on a Typhoon FLA 9500 with Control Software v. 1.0 (GE Healthcare).

For LC-MS/MS analysis, ADP-ribosylation was performed with 200 nM DNA substrate, 2 mM NAD^+^, 2 µM recombinant PARP1, and 20 µM olaparib or 100 nM pentostatin (Sigma) if indicated in ADPR buffer for 60 min at 37 °C followed by phenol/chloroform extraction and ethanol precipitation. PARG treatment was done with 200 nM in vitro PARylated DNA substrate and 0.2 ng/µl PARG in PARG buffer for 2 h at 30 °C. To remove free PAR chains PARG treatment was followed by DNA cleanup with DNA clean and concentrator 5 (Zymo Research) according to manufacturer’s instructions. Control reactions were done with PARP1 or PARG heat-denatured at 70 °C for 10 min.

### Statistics and reproducibility

Data presented as bar diagrams are displayed as arithmetic mean, error bars represent standard deviation of the indicated replicates with propagation of error if required after data normalization. Statistical analysis was carried out with Student’s *t* test as indicated in the figure legends using Microsoft Excel 2016. For multiple comparisons adjusted *p* values were calculated by Dunnett’s test using GraphPad Prism v. 9. Significances are displayed in the respective figure panels; n.s., not significant. Dot blots, SDS-PAGE gels, southwestern blots, autoradiographs and LC-MS/MS electropherograms are representative of at least three independent experiments with similar outcomes.

### Reporting summary

Further information on research design is available in the Nature Research Reporting Summary linked to this article.

## Supplementary information

Supplementary information

Reporting Summary

## Data Availability

All data are available from the corresponding authors upon request. Source data are provided with this paper.

## Source data

Source Data

## References

[1] Brady, P. N., Goel, A. & Johnson, M. A. Poly(ADP-Ribose) polymerases in host-pathogen interactions, inflammation, and immunity. *Microbiol. Mol. Biol. Rev.* 83; 10.1128/MMBR.00038-18 (2019). 10.1128/MMBR.00038-18 PMC6383445 30567936

[2] Hottiger MO (2015). Nuclear ADP-ribosylation and its role in chromatin plasticity, cell differentiation, and epigenetics. Annu. Rev. Biochem..

[3] Gupte R, Liu Z, Kraus WL (2017). PARPs and ADP-ribosylation: recent advances linking molecular functions to biological outcomes. Genes Dev..

[4] Takamura-Enya T (2001). Mono(ADP-ribosyl)ation of 2’-deoxyguanosine residue in DNA by an apoptosis-inducing protein, pierisin-1, from cabbage butterfly. Proc. Natl. Acad. Sci. USA.

[5] Nakano T (2006). Purification and molecular cloning of a DNA ADP-ribosylating protein, CARP-1, from the edible clam Meretrix lamarckii. Proc. Natl. Acad. Sci. USA.

[6] Lyons B (2016). Scabin, a novel DNA-acting ADP-ribosyltransferase from Streptomyces scabies. J. Biol. Chem..

[7] Nakano T, Takahashi-Nakaguchi A, Yamamoto M, Watanabe M (2015). Pierisins and CARP-1: ADP-ribosylation of DNA by ARTCs in butterflies and shellfish. Curr. Top. Microbiol. Immunol..

[8] Jankevicius G, Ariza A, Ahel M, Ahel I (2016). The toxin-antitoxin system DarTG catalyzes reversible ADP-ribosylation of DNA. Mol. Cell.

[9] Schuller M (2021). Molecular basis for DarT ADP-ribosylation of a DNA base. Nature.

[10] Matta E, Kiribayeva A, Khassenov B, Matkarimov BT, Ishchenko AA (2020). Insight into DNA substrate specificity of PARP1-catalysed DNA poly(ADP-ribosyl)ation. Sci. Rep..

[11] Zarkovic G (2018). Characterization of DNA ADP-ribosyltransferase activities of PARP2 and PARP3: new insights into DNA ADP-ribosylation. Nucleic Acids Res..

[12] Belousova EA, Ishchenko A, Lavrik OI (2018). Dna is a new target of Parp3. Sci. Rep..

[13] Talhaoui I (2016). Poly(ADP-ribose) polymerases covalently modify strand break termini in DNA fragments in vitro. Nucleic Acids Res..

[14] Munnur D, Ahel I (2017). Reversible mono-ADP-ribosylation of DNA breaks. FEBS J..

[15] Kawamitsu H (1984). Monoclonal antibodies to poly(adenosine diphosphate ribose) recognize different structures. Biochemistry.

[16] James DI (2016). First-in-class chemical probes against poly(ADP-ribose) glycohydrolase (PARG) inhibit DNA repair with differential pharmacology to olaparib. ACS Chem. Biol..

[17] Menear KA (2008). 4-3-(4-cyclopropanecarbonylpiperazine-1-carbonyl)−4-fluorobenzyl-2H-phthalazin-1-one: a novel bioavailable inhibitor of poly(ADP-ribose) polymerase-1. J. Med. Chem..

[18] Munnur D (2019). Reversible ADP-ribosylation of RNA. Nucleic Acids Res..

[19] Martello R, Mangerich A, Sass S, Dedon PC, Bürkle A (2013). Quantification of cellular poly(ADP-ribosyl)ation by stable isotope dilution mass spectrometry reveals tissue- and drug-dependent stress response dynamics. ACS Chem. Biol..

[20] Alvarez-Gonzalez R, Jacobson MK (1987). Characterization of polymers of adenosine diphosphate ribose generated in vitro and in vivo. Biochemistry.

[21] Hottiger MO, Hassa PO, Lüscher B, Schüler H, Koch-Nolte F (2010). Toward a unified nomenclature for mammalian ADP-ribosyltransferases. Trends Biochem. Sci..

[22] Ray Chaudhuri A, Nussenzweig A (2017). The multifaceted roles of PARP1 in DNA repair and chromatin remodelling. Nat. Rev. Mol. Cell Biol..

[23] Sutcu, H. H., Matta, E. & Ishchenko, A. A. Role of PARP-catalyzed ADP-ribosylation in the crosstalk Between DNA strand breaks and epigenetic regulation. *J. Mol. Biol.*10.1016/j.jmb.2019.12.019 (2019). 10.1016/j.jmb.2019.12.019 31866292

[24] Iacovoni JS (2010). High-resolution profiling of gammaH2AX around DNA double strand breaks in the mammalian genome. EMBO J..

[25] Alseth I, Dalhus B, Bjørås M (2014). Inosine in DNA and RNA. Curr. Opin. Genet. Dev..

[26] Karran P, Lindahl T (1980). Hypoxanthine in deoxyribonucleic acid: generation by heat-induced hydrolysis of adenine residues and release in free form by a deoxyribonucleic acid glycosylase from calf thymus. Biochemistry.

[27] Barlow T, Ding J, Vouros P, Dipple A (1997). Investigation of hydrolytic deamination of 1-(2-hydroxy-1-phenylethyl)adenosine. Chem. Res. Toxicol..

[28] Begemann P (2011). Identification and characterization of 2’-deoxyadenosine adducts formed by isoprene monoepoxides in vitro. Chem. Res. Toxicol..

[29] Stachowicz-Kuśnierz A, Korchowiec J (2016). Nucleophilic properties of purine bases: inherent reactivity versus reaction conditions. Struct. Chem..

[30] Lonskaya I (2005). Regulation of poly(ADP-ribose) polymerase-1 by DNA structure-specific binding. J. Biol. Chem..

[31] Gibbs-Seymour I, Fontana P, Rack JGM, Ahel I (2016). HPF1/C4orf27 is a PARP-1-interacting protein that regulates PARP-1 ADP-ribosylation activity. Mol. Cell.

[32] Bonfiglio JJ (2017). Serine ADP-ribosylation depends on HPF1. Mol. Cell.

[33] Rack JGM, Palazzo L, Ahel I (2020). (ADP-ribosyl) hydrolases: structure, function, and biology. Genome Res..

[34] Houl JH (2019). Selective small molecule PARG inhibitor causes replication fork stalling and cancer cell death. Nat. Commun..

[35] Dawlaty MM (2014). Loss of Tet enzymes compromises proper differentiation of embryonic stem cells. Dev. Cell.

[36] Amé J-C, Héberlé É, Camuzeaux B, Dantzer F, Schreiber V (2017). Purification of recombinant human PARG and activity assays. Methods Mol. Biol..

[37] Langelier M-F, Planck JL, Servent KM, Pascal JM (2011). Purification of human PARP-1 and PARP-1 domains from Escherichia coli for structural and biochemical analysis. Methods Mol. Biol..

[38] Yunusov D (2009). Kinetic capillary electrophoresis-based affinity screening of aptamer clones. Anal Chim. Acta.

