# Supplementary material for: Mammalian N1-adenosine PARylation is a reversible DNA modification
Source: Nat Commun. 2022 Oct 17;13:6138. doi: 10.1038/s41467-022-33731-w (PMC9576699; doi:10.1038/s41467-022-33731-w)
Supplement: Supplementary file 1 — Supplementary information [file 41467_2022_33731_MOESM1_ESM.pdf]

Supplementary information

Mammalian N1-adenosine PARylation is a reversible DNA modification

Michael U. Musheev[1], Lars Schomacher[1*], Amitava Basu[1], Dandan Han[1, 3], Laura Krebs[1], Carola Scholz[1], and Christof Niehrs[1,2*]

[1] Institute of Molecular Biology (IMB), 55128 Mainz, Germany

[2] Division of Molecular Embryology, DKFZ-ZMBH Alliance, 69120 Heidelberg, Germany

[3] Present address: STEMCELL Technologies Germany GmbH, 50933 Cologne, Germany

These authors contributed equally: Michael U. Musheev, Lars Schomacher, Amitava Basu

[*] Corresponding authors: l.schomacher@imb-mainz.de; c.niehrs@imb-mainz.de

Supplementary Figures

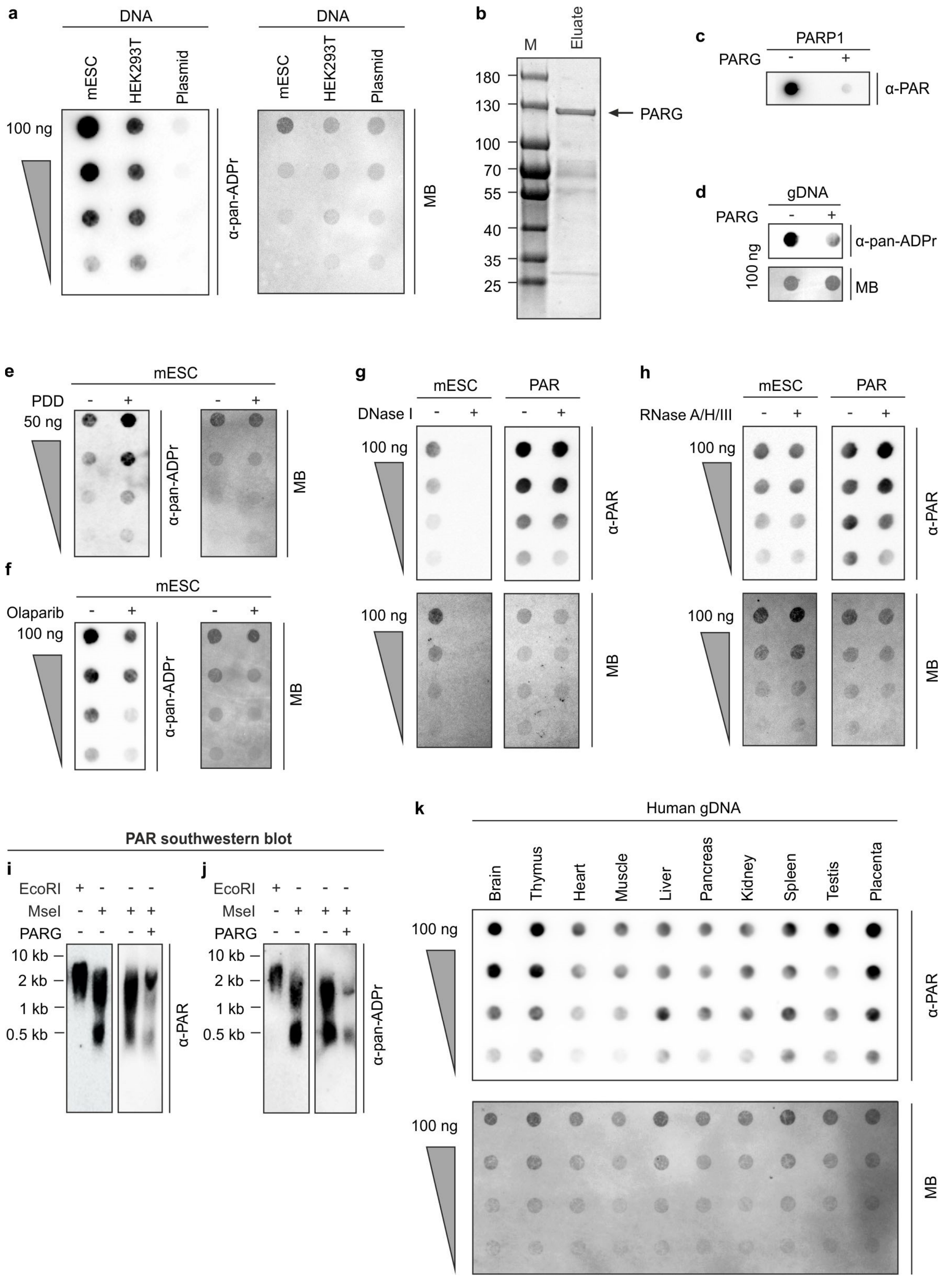

Supplementary Fig. 1: DNA PARylation in mouse and human tissues.

a, Dot blot analysis for PARylation of mESC and HEK293T serially (2x) diluted genomic DNA as in Fig. 1a but using anti-pan-ADP-ribose binding reagent. Plasmid DNA served as negative control. MB, methylene blue staining.

b, SDS-PAGE analysis of the purified full-length PAR glycohydrolase (PARG[36], arrow) used in the study. Calculated molecular weight for PARG is 112 kDa. Molecular weight of marker proteins (M) is indicated on the left in kDa. Eluate, supernatant post treatment with PreScission protease. The analysis is representative of three independent purifications with similar outcomes.

c-h, Dot blot analysis for PARylation employing either anti-poly (ADP-ribose) mouse monoclonal antibody (α-PAR) or anti-pan-ADP-ribose binding reagent (α-pan-ADPr) of (**c**) autoPARylated PARP1 treated with PARG as indicated, (**d**) mESC DNA treated with PARG, (**e**) DNA purified from mESCs treated with the PARG inhibitor PDD00017273, (**f**) DNA purified from mESCs treated with the PARP inhibitor olaparib, (**g**) genomic mESC DNA (left) and PAR polymer (right) treated with DNase I, (**h**) genomic mESC DNA (left) and PAR polymer (right) treated with RNase A, RNase H and RNase III, MB, methylene blue.

i-j, Southwestern blot analysis for PARylation of HEK293T DNA treated with EcoRI, MseI, and PARG as indicated and utilizing anti-poly (ADP-ribose) mouse monoclonal antibody (α-PAR, **i**) or anti-pan-ADP-ribose binding reagent (α-pan-ADPr, **j**). Length of marker DNA is shown on the left. Southwesterns are representative of three independent experiments with similar outcomes.

k, Dot blot analysis for PARylation of DNA from the indicated adult human tissues. gDNA was serially diluted (2x). MB, methylene blue.

Source data are provided as a Source Data file.

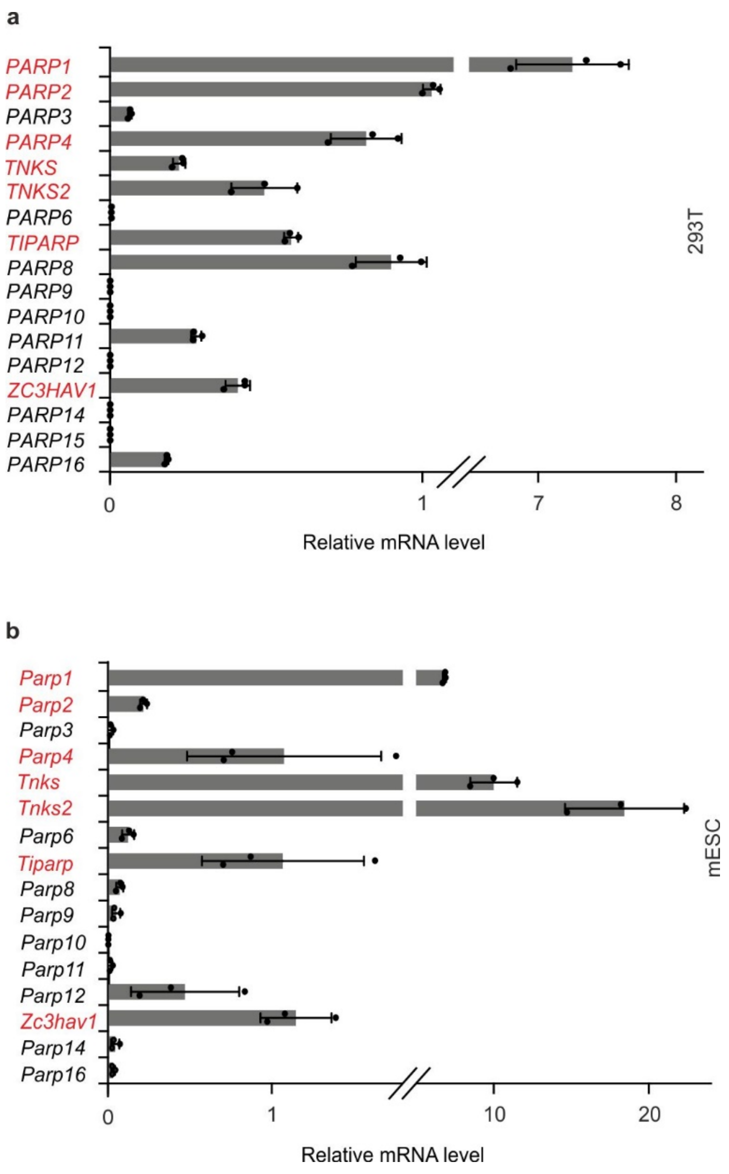

Supplementary Fig. 2: PARP candidates for DNA PARylation selected by their expression levels.

a, Expression analysis of *PARP* genes in 293T cells. *PARP genes* commonly high expressed in 293T and mESCs are highlighted in red. Data are presented as mean values +/- s.d. of 3 biological replicates.

b, Expression analysis of *Parp* genes in mESC. *Parp genes* commonly high expressed in 293T and mESCs are highlighted in red. Data are presented as mean values +/- s.d. of 3 biological replicates.

Source data are provided as a Source Data file.

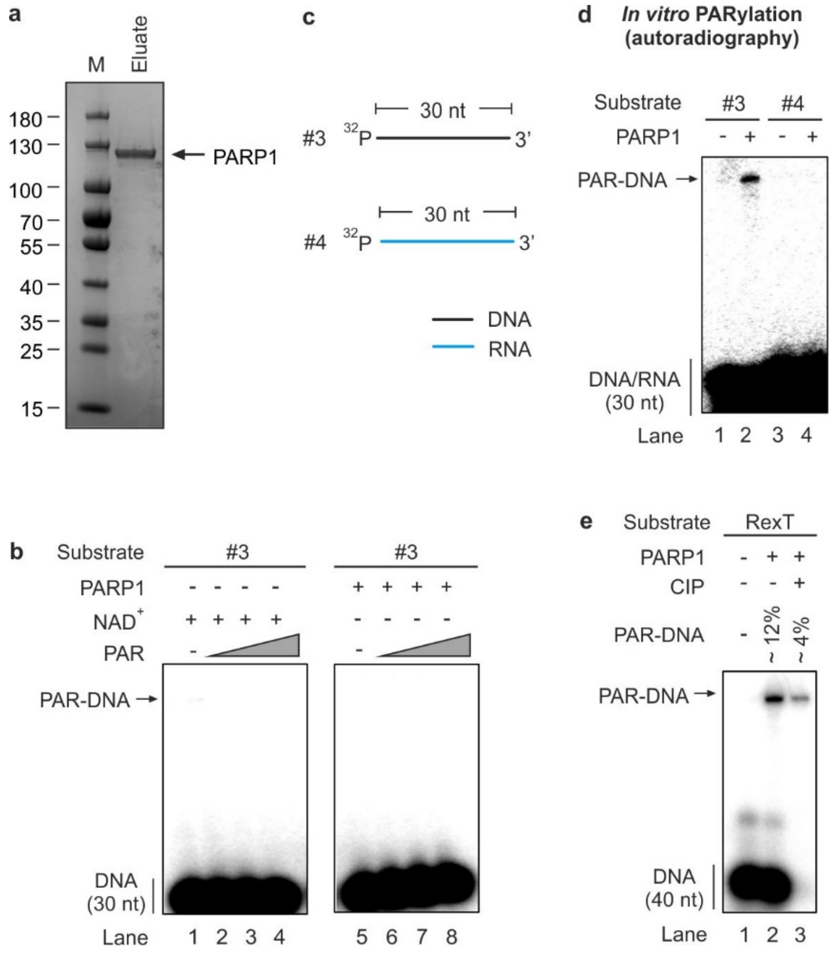

Supplementary Fig. 3: PARP1 preferentially PARylates ssDNA *in vitro*.

a, SDS-PAGE analysis of purified full-length PARP1 (arrow) used in the study. Calculated molecular weight for PARP1 is 113 kDa. Molecular weight of marker proteins (M) are indicated on the left in kDa. Eluate, PARP1 pooled fraction from gel filtration chromatography. Analysis is representative of three independent purifications with similar outcomes.

b, Free PAR chains are not conjugated to DNA. Substrate #3 was incubated with increasing amounts of free PAR chains in presence of NAD^+ or PARP1 as indicated followed by denaturing PAGE and autoradiography of the reaction products. Note the absence of PARylated DNA products, indicating that free PAR chains are not attached to substrate ssDNA either spontaneously or by PARP1. Autoradiography is representative of three independent experiments with similar outcomes.

c, Scheme of ssDNA and ssRNA substrates used for *in vitro* PARylation assays. The 30 nt DNA strand is designated as standard oligo in the main text. ^{32}P, 5'-phosphate with ^{32}P-label.

d-e, Denaturing PAGE and autoradiography of reaction products from PARylation assays in presence of PARP1, with post reaction CIP-treatment and substrates as indicated. (**d**), PARP1 does not PARylate ssRNA. (**e**), PARP1 PARylates ssDNA RexT both internally (8%) and terminally (4%). Autoradiographies are representative of three independent experiments with similar outcomes.

Source data are provided as a Source Data file.

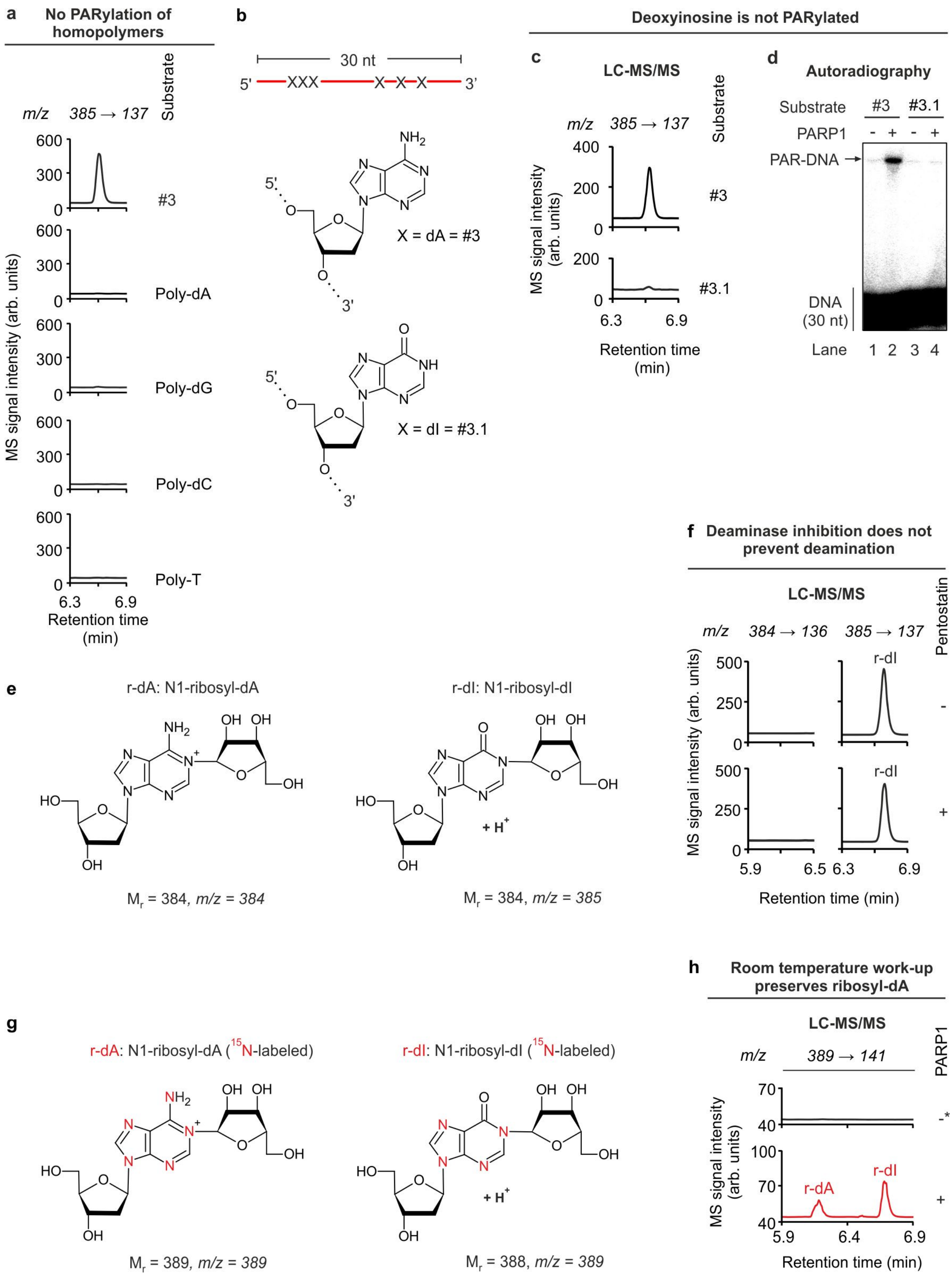

a No PARylation of homopolymers
m/z 385 → 137
Substrate
MS signal intensity (arb. units)
#3
Poly-dA
Poly-dG
Poly-dC
Poly-T
Retention time (min)
b
30 nt
5' XXX X X X 3'
X = dA = #3
X = dI = #3.1
Deoxyinosine is not PARylated
c LC-MS/MS
m/z 385 → 137
Substrate
MS signal intensity (arb. units)
#3
#3.1
Retention time (min)
d Autoradiography
Substrate #3 #3.1
PARP1 - + - +
PAR-DNA
DNA (30 nt)
Lane 1 2 3 4
e
r-dA: N1-ribosyl-dA
r-dI: N1-ribosyl-dI
Mr = 384, m/z = 384
Mr = 384, m/z = 385
f Deaminase inhibition does not prevent deamination
LC-MS/MS
m/z 384 → 136 385 → 137
Pentostatin
MS signal intensity (arb. units)
r-dI
Retention time (min)
g
r-dA: N1-ribosyl-dA (15N-labeled)
r-dI: N1-ribosyl-dI (15N-labeled)
Mr = 389, m/z = 389
Mr = 388, m/z = 389
h Room temperature work-up preserves ribosyl-dA
LC-MS/MS
m/z 389 → 141
PARP1
MS signal intensity (arb. units)
-*
r-dA
r-dI
Retention time (min)

Supplementary Fig. 4: Identification of the PARylated base by LC-MS/MS analyses.

a, LC-MS/MS chromatograms of reaction products from *in vitro* PARylation assays with the indicated substrates and human PARP1. Mass transition corresponds to shift expected for loss of a deoxyribose + ribose (*m/z* 385 → 137) from the parental molecule 'nucleoside 385'.

b, Scheme of the 30mer ssDNA standard oligo used for *in vitro* PARylation assays. In substrate #3 'X' represents dA, in substrate #3.1 'X' is dI. Structures of dA and dI are shown below.

c, LC-MS/MS chromatogram for 'nucleoside 385' in reaction products from PARylation assay with the substrates indicated.

d, Autoradiography of denaturing PAGE of reaction products from PARylation assay with the indicated substrates. Autoradiography is representative of three independent experiments with similar outcomes.**e**, Structure, relative molecular weight and *m/z* ratio for N1-ribosyl-dA (left) and N1-ribosyl-dI (right).

f, LC-MS/MS chromatograms of reaction products from *in vitro* PARylation assay in presence of substrate #3 and human PARP1. PAR reaction and enzymatic degradation of reaction products for mass spec analysis was done in presence or absence of the deaminase inhibitor Pentostatin as indicated. Products were scanned for signals with *m/z* transitions expected for N1-ribosyl-dA (r-dA, 384 → 136, left) or N1-ribosyl-dI (r-dI, 385 → 137, right)

g, Structure, relative molecular weight and *m/z* ratio for ^{15}N-labeled N1-ribosyl-dA (left) and ^{15}N-labeled N1-ribosyl-dI (right). Heavy isotope labeled nitrogens are highlighted in red.

h, LC-MS/MS chromatograms of reaction products from *in vitro* PARylation assay with the $^{15}N_5$-dA-labeled 83mer ssDNA oligo in presence of native and denatured (-*) PARP1 as indicated. Unlike for standard protocol, sample denaturation at 95 °C was omitted before mass spec analysis to avoid deamination. Samples were screened for signals with the same *m/z* transition of 389 → 141 as expected for both $^{15}N_5$-labeled N1-ribosyl-dA and $^{15}N_4$-labeled N1-ribosyl-dI, but distinguished by different retention times.

Source data are provided as a Source Data file.

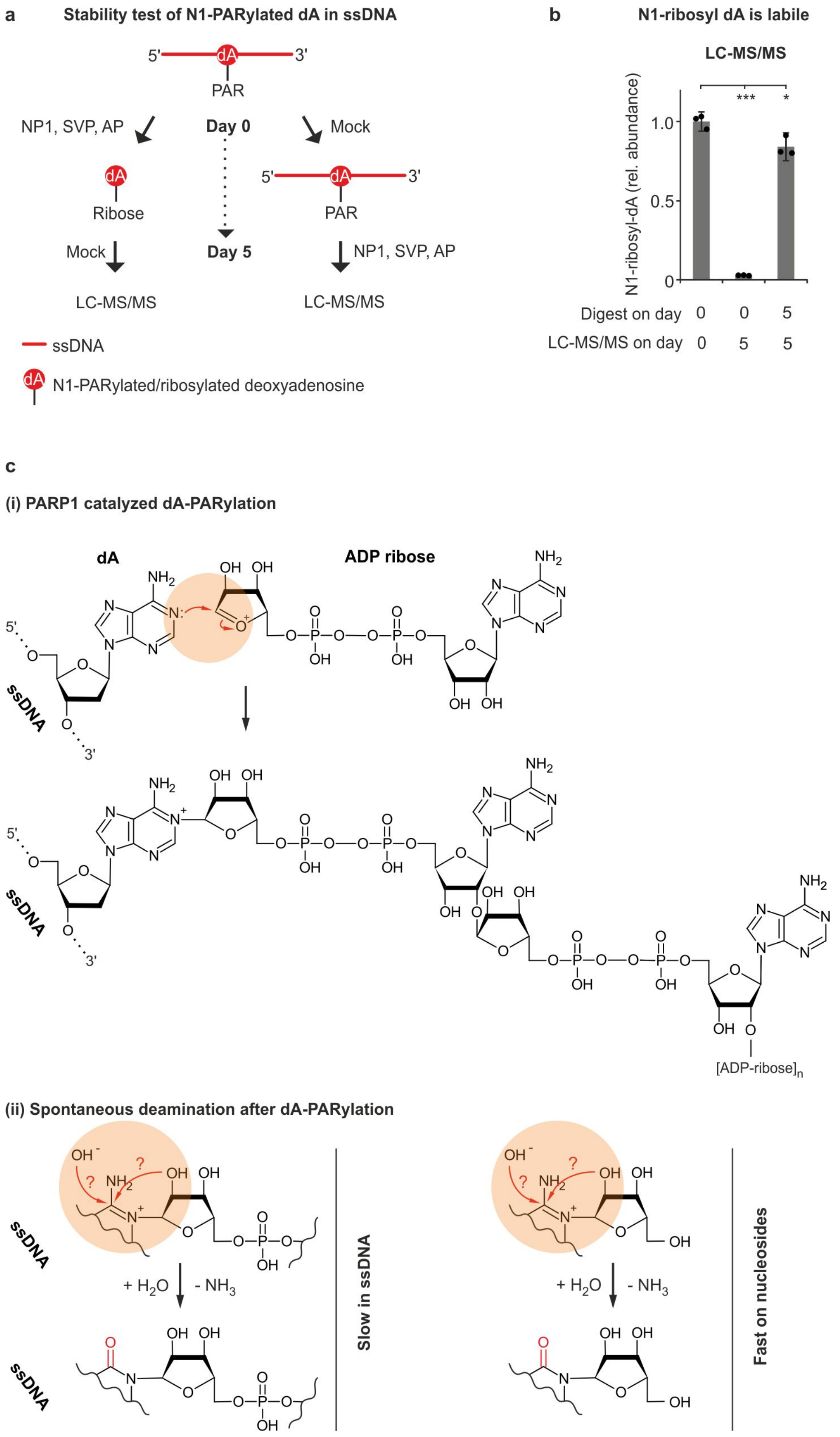
a
Stability test of N1-PARylated dA in ssDNA
5'
dA
3'
PAR
NP1, SVP, AP
Day 0
Mock
Ribose
Day 5
Mock
NP1, SVP, AP
LC-MS/MS
LC-MS/MS
ssDNA
N1-PARylated/ribosylated deoxyadenosine
b
N1-ribosyl dA is labile
LC-MS/MS
N1-ribosyl-dA (rel. abundance)
***
*
1.0
0.5
0
Digest on day 0 0 5
LC-MS/MS on day 0 5 5
c
(i) PARP1 catalyzed dA-PARylation
dA
ADP ribose
ssDNA
[ADP-ribose]n
(ii) Spontaneous deamination after dA-PARylation
+ H2O
- NH3
Slow in ssDNA
Fast on nucleosides

Supplementary Fig. 5: Stability of PARylated-dA in ssDNA and proposed reaction mechanisms.

a, Outline of stability test for PARylated dA within ssDNA. An *in vitro* PARylated 83mer oligonucleotide is either degraded to single nucleosides by combined treatment with nuclease P1, snake venom phosphodiesterase and alkaline phosphatase (NP1, SVP, AP) or mock-treated on day 0. Samples are stored at 4 °C for 5 days followed by mock treatment of single nucleosides and degradation of the 83mer with NP1, SVP, AP and subsequent LC-MS/MS analysis.

b, LC-MS/MS quantification of N1-ribosyl-dA in samples as described in a. As reference, the PARylated 83mer was degraded and analyzed on day 0, and arbitrarily set to 1. Data are presented as mean values +/- s.d. of 3 independent experiments; indicated p-values as by two-sided Dunnett's test. Note, while we readily detect a signal for N1-ribosyl-dA on samples that were kept non-degraded for 5 days, the signal was erased in samples that were degraded immediately and stored for 5 days at -20°C. Moreover, samples that were degraded and analyzed directly after *in vitro* PARylation show marginally higher (~20%) N1-ribosyl-dA signals compared to samples stored for 5 days and digested only before LC-MS/MS. Hence, deamination is slow on PARylated-dA in intact DNA but vastly accelerates on ribosylated dA-nucleosides.

c, Proposed reaction mechanisms. (i), PARP1 catalyzes a nucleophilic attack of N1 from dA to C1 of the oxonium ion of ADP ribose, followed by PARylation. (ii), Spontaneous deamination is slow on PARylated dA within ssDNA but fast on ribosyl-dA nucleosides, and presumably proceeds via hydrolytic attack at C6 by free hydroxyl ions or hydroxyl groups of the attached ribose as indicated by two alternative arrows.

Source data are provided as a Source Data file.

Supplementary Tables

SupplementaryTable 1: Oligonucleotides used in this study.

PAR-DIP- and ChIP-qPCR primers (IDT) and UPL probe numbers (Roche)

**Target**	**Direction**	**Sequence (5’→3‘)**	**Probe #**
human Chr1: 88992918	Forward<br>Reverse	CACCCCTCATCCAGTCTCTT<br>ATTGACTCTGGGGTTTGCAC	162
human Chr6: 89638472	Forward<br>Reverse	CAGCTCAGACCCAGGTAAGC<br>GGTGGACATGGCTGCTTC	13
human Chr22: 20496025	Forward<br>Reverse	CCTCGGTCCTACCCTCATTC<br>CGGGGAGGGAGATAGTGAG	51
human Chr1: 3186484	Forward<br>Reverse	CCTGGGATGGAGGACTCTC<br>GCCCCTGTCTACATTCCTGA	3

RT-qPCR primers (IDT) and UPL probe numbers (Roche)

**Gene**	**Direction**	**Sequence (5’→3‘)**	**Probe #**
mouse *Gapdh*	Forward<br>Reverse	AGCTTGTCATCAACGGGAAG<br>TTTGATGTTAGTGGGGTCTCG	9
mouse *Parp1*	Forward<br>Reverse	AGGCCGCCTACTCTATCCTC<br>GATTCACTGCTGCCTTGAGA	40
mouse *Parp2*	Forward<br>Reverse	TCGTCCTTCAAGAGCGATG<br>CCAAGAATTACTGCACTATGAAGATG	97
mouse *Parp3*	Forward<br>Reverse	TCAGTGCTGTGCAATAGACTCTTA<br>GGGGCTTAGGAAGATGTGTG	2
mouse *Parp4*	Forward<br>Reverse	TGCAACAGCGCAGTCTCTTA<br>TGCTCCACAGCTTTCAGTTG	34
mouse *Tnks (Parp5a)*	Forward<br>Reverse	CCCACACACAAAGACAGATCA<br>GCCATTTTCATGGTGCTGA	4
mouse *Tnk2 (Parp5b)*	Forward<br>Reverse	TCCAATTCACAAAGACAGATCG<br>TCCCAAGGTTACTCGACAAAA	27
mouse *Parp6*	Forward<br>Reverse	GGCTACGGTTTTCTCTCTCTCA<br>CCTTATTCGATGGCTGGAAA	21
mouse *Tiparp (Parp7)*	Forward<br>Reverse	CAGAACAGGGGGTTCCAAT<br>CCACTGTCCCACTGATGGTT	31
mouse *Parp8*	Forward	CGGTATTTTAACACCATCATCG	75

	Reverse	GGAATGCTTTTTGCACCATT	
mouse *Parp9*	Forward<br>Reverse	TCAGCGGCTGGACTCATAC<br>GAGAGTGCGGTCCACAGAC	58
mouse *Parp10*	Forward<br>Reverse	GAACCCTTCTAAGGGCAGGT<br>GGATCCTAATTGATCCCGAGT	1
mouse *Parp11*	Forward<br>Reverse	GGCGTTGGGAGTTCAGAGT<br>CAAACAAACAAACAAACAAAAACA	2
mouse *Parp12*	Forward<br>Reverse	AAAGGGCTTACTTGGCATTCT<br>TGCCATACACAAGGTTTTTCTG	1
mouse *Zc3hav1*<br>*(Parp13)*	Forward<br>Reverse	AGGTATTCTGTTTCATCACCAAGA<br>CTTCGGGGAGGCTGATCT	3
mouse *Parp14*	Forward<br>Reverse	TGTGAAACAAGGTGATTTGGA<br>GGGACATCTTCTGTCTTTTCTGA	3
mouse *Parp16*	Forward<br>Reverse	GCCAAATAGAGGGGGAGATG<br>GCTTGGACCCTTCTCCATAA	1
human *TBP*	Forward<br>Reverse	GAACATCATGGATCAGAACAACA<br>ATAGGGATTCCGGGAGTCAT	87
human *PARP1*	Forward<br>Reverse	ACTTCCTCCAGGACGTCTCC<br>TGTGCGCTAAGAACAACTCC	22
human *PARP2*	Forward<br>Reverse	ACCAAGAAAGCCCCACTTG<br>AGCCCGAATACAATCCTCAA	59
human *PARP3*	Forward<br>Reverse	AACTGGGTAATCGGAAGCTG<br>ATGATGCGGAGCCCACTA	49
human *PARP4*	Forward<br>Reverse	GAAGTGAACCTGGGACTATTGG<br>TCACAGACATTAACCATGTCTCTTATT	53
human *TNKS (PARP5A)*	Forward<br>Reverse	GGCAAACGTAAATGCAAAGG<br>AACATCCTTCCTTCCAAAACCT	7
human *TNKS2*<br>*(PARP5B)*	Forward<br>Reverse	GGGTTGTTTAGCCAGAGTGAA<br>TCAGCTCCGTGTTGTAACAAAT	2
human *PARP6*	Forward<br>Reverse	GCCCCATCTCCAGTATTTCC<br>CTCATCCTTGGAGGGCATC	56
human *TIPARP (PARP7)*	Forward<br>Reverse	GGAAATTCTTCTGTAGGGACCA<br>AATCAATCGAATGACAGACTCG	58
human *PARP8*	Forward<br>Reverse	ATTTGGATTGGGACATCAGC<br>ATTGTGCAGGCAATTGGATT	86
human *PARP9*	Forward	GAACAACCTGACCCTCCAGA<br>TCATGTGGGTTTACAGAATTAACAA	81

	Reverse		
human *PARP10*	Forward<br>Reverse	CCTTCTACGACACCCTGGAC<br>CCGGTACAGCTCATACTGCTG	89
human *PARP11*	Forward<br>Reverse	CCCCCTTTTCTATCAGTGCTT<br>TGCAGAGGAATAAGCTGATATGG	14
human *PARP12*	Forward<br>Reverse	GTCCCACAGGGGACTTCTG<br>TTTTCCGGATATGGTACAAACA	63
human *ZC3HAV1 (PARP13)*	Forward<br>Reverse	AGACCAACATTTGTGCCTCA<br>CACTGACGAGGTCTTTGCTG	84
human *PARP14*	Forward<br>Reverse	GTGTTCTTCTACCCGGAGGA<br>TTCCTTGCCATACCAACTCA	29
human *PARP15*	Forward<br>Reverse	CACCCGAACTTGTTCTTCCTA<br>CCATTTGCCTTTTCTTTACCTG	53
human *PARP16*	Forward<br>Reverse	CAGCTGCTGCGAGTGAAG<br>CAGGAGAGCTGGCTCGAA	79

siRNA sequences (siGENOME SMARTpool, mouse and human, Dharmacon)

**Name**	**Target sequence**
siControl	1: UAGCGACUAAACACAUCAA<br>2: UAAGGCUAUGAAGAGAUAC<br>3: AUGUAUUGGCCUGUAUUAG<br>4: AUGAACGUGAAUUGCUCAA
human si*PARP1*	1: GAAAGUGUGUUCAACUAAU<br>2: GCAACAAACUGGAACAGAU<br>3: GAAGUCAUCGAUAUCUUUA<br>4: GAUAGAGCGUGAAGGCGAA
human si*PARP2*	1: AAGGAUUGCUUCAAGGUAA<br>2: ACAGCUAGAUCUUCGGGUA<br>3: GCCAGAGACAGGAGUCGAA<br>4: ACAAUUGGGAAGAUCGAGA
human si*PARP4*	1: CAACUGAACCACUAUUUAA<br>2: GAGCAGUUCUGAAGUGAAA<br>3: GUGCACACAUAUAAUCUUA<br>4: CCACAGACUUUGAGGAUGA
human si*TNKS (PARP5A)*	1: GAACAGAGAUGGAAAUACA<br>2: CAGAGUAUCUUAUCACUUA

	3: GGAAGUAGCUGAAUAUCUU<br>4: CAACAGAGUUCGAAUAGUU
human si*TNKS2 (PARP5B)*	1: GGAAAGACGUAGUUGAAUA<br>2: UAGCAUAACUCAAUUCGUA<br>3: AGACAGAUCUUGUUACAUU<br>4: AAUGUAAAUUGCCGCGAUA
human si*TIPARP (PARP7)*	1: GGCCGUGACAGGAUAAUAA<br>2: GAAAGAGGUUCGAUUUAUG<br>3: AAGGCAAGCUACUCUCAUA<br>4: GGCAGAGUGUAUUCAAUGA
human si*ZC3HAV1 (PARP13)*	1: GCACAUGGAUUCAGUAUGG<br>2: GCAAGCACAUGCAGAAGAA<br>3: GAACAAAGAGGAAUUAGCA<br>4: CAAAUAUUCUCAUGAGGUU
mouse si*Parp1*	1: GGAGGAAGGUGUCAACAAA<br>2: UAAAGAAGCUGACGGUGAA<br>3: CAAAGUAUCCCAAGAAGUU<br>4: GAAAUAUCCUACCUCAAGA
mouse si*Parp2*	1: GGGAAAGGCUCAUGUGUAU<br>2: GAAGGCGAGUGCUAAAUGA<br>3: GCAAGAAGAUGCGCACGUG<br>4: GGACUAUACUAUGACCUUG

DNA and RNA oligonucleotides used for *in vitro* PARylation (IDT and Sigma)

**Name**	**Sequence (5’→3‘)**
30mer DNA_up (#3)	TGTGCAAACGCCGGCTTCCGCACAGAGGCC
30mer RNA_low (#4)	GGCCUCUGUGCGGAAGCCGGCGUUUGCACA
30mer DNA_low	GGCCTCTGTGCGGAAGCCGGCGTTTGCACA
40mer DNA_low	CACGCAAGGTGGCCTCTGTGCGGAAGCCGGCGTTTGCACA
30mer DNA_up_2’d-inosine (#3.1)	TGTGC[dI][dI][dI]CGCCGGCTTCCGC[dI]C[dI]G[dI]GGCC
RexT	GGAATTCCCCGCGCCAAATTTCTCTAAGTCTCCGCGCCAC
83mer DNA	CTCCTCTGACTGTAACCACGCCGGTACGTTACGATACGATTA<br>CGTAATACGATTTCGAACCGGCATAGGTAGTCCAGAAGCCT
Poly-dA	AAAAAAAAAAAAAAAAAAAA

Poly-dG	GGGGGGGGGGGGGGGGGGGG
Poly-dC	CCCCCCCCCCCCCCCCCCCC
Poly-T	TTTTTTTTTTTTTTTTTTTT

dI, 2'd-inosine

<u>DNA oligonucleotides (IDT) used for asymmetric PCR</u>

**Purpose**	**Direction**	**Sequence (5'→3')**
Primer	Forward	CTCCTCTGACTGTAACCACG
	Reverse	AGGCTTCTGGACTACCTATGC
Template		CTCCTCTGACTGTAACCACGCCGGTACGTTACGATACGATTA CGTAATACGATTTCGAACCGGCATAGGTAGTCCAGAAGCCT

SupplementaryTable 2: MRM transitions used in this study.

Nucleoside	Precursor ion (*m/z*)	Fragment ion (*m/z*)	Collision energy	Cell accelerator voltage
dA	252	136	6	8
$^{15}N_5{}^{13}C_{10}$-dA	267	146	6	8
dG natural isotopologue (+1)	269	153	6	5
$^{15}N_5$-dG	273	157	6	5
R-Ado (1)(qualitative)	400	136	6	8
R-Ado (2)(quantitative)	400	268	5	8
$^{15}N_5$- R-Ado	405	141	6	8
2R-Ado	532	136	6	8
$^{15}N_5$- 2R-Ado	537	141	6	8
N1-ribosyl-dI (‘nucleoside 385’) (1)	385	137	17	5
N1-ribosyl-dI (‘nucleoside 385’) (2)	385	269	1	3
N1-ribosyl-dA	384	137	17	5
$^{15}N_1$-‘nucleoside 385’	386	138	17	5
$^{15}N_2$-‘nucleoside 385’	387	139	17	5
$^{15}N_3$-‘nucleoside 385’	388	140	17	5
$^{15}N_4$-ribosyl-dI, $^{15}N_5$-ribosyl-dA	389	141	17	5
$^{15}N_5$-‘nucleoside 385’	390	142	17	5
$^{13}C_{10}$-ribosyl-dI	395	142	17	5

MS 1 and 2 resolutions were set to unit for all ions. Note, all quantification were performed using stable-isotope dilution LC-MS/MS except for N1-ribosyl-dA or N1-ribosyl-dI due to lack of the respective isotopologue.
