# Supplementary material for: Mammalian N1-adenosine PARylation is a reversible DNA modification
Source: Nat Commun. 2022 Oct 17;13:6138. doi: 10.1038/s41467-022-33731-w (PMC9576699; doi:10.1038/s41467-022-33731-w)
Supplement: Supplementary file 2 — Reporting Summary [file 41467_2022_33731_MOESM2_ESM.pdf]

nature portfolio

Corresponding author(s): Christof Niehrs; Lars Schomacher

Last updated by author(s): Sep 19, 2022

Reporting Summary

Nature Portfolio wishes to improve the reproducibility of the work that we publish. This form provides structure for consistency and transparency in reporting. For further information on Nature Portfolio policies, see our Editorial Policies and the Editorial Policy Checklist.

Statistics

For all statistical analyses, confirm that the following items are present in the figure legend, table legend, main text, or Methods section.

n/a	Confirmed	
☐	☒	The exact sample size (*n*) for each experimental group/condition, given as a discrete number and unit of measurement
☐	☒	A statement on whether measurements were taken from distinct samples or whether the same sample was measured repeatedly
☐	☒	The statistical test(s) used AND whether they are one- or two-sided<br>*Only common tests should be described solely by name; describe more complex techniques in the Methods section.*
☒	☐	A description of all covariates tested
☐	☒	A description of any assumptions or corrections, such as tests of normality and adjustment for multiple comparisons
☐	☒	A full description of the statistical parameters including central tendency (e.g. means) or other basic estimates (e.g. regression coefficient) AND variation (e.g. standard deviation) or associated estimates of uncertainty (e.g. confidence intervals)
☐	☒	For null hypothesis testing, the test statistic (e.g. *F*, *t*, *r*) with confidence intervals, effect sizes, degrees of freedom and *P* value noted<br>*Give P values as exact values whenever suitable.*
☒	☐	For Bayesian analysis, information on the choice of priors and Markov chain Monte Carlo settings
☒	☐	For hierarchical and complex designs, identification of the appropriate level for tests and full reporting of outcomes
☒	☐	Estimates of effect sizes (e.g. Cohen's *d*, Pearson's *r*), indicating how they were calculated

Our web collection on statistics for biologists contains articles on many of the points above.

Software and code

Policy information about availability of computer code

Data collection	Agilent MassHunter Workstation v. B06.00 and B09.00; Leica Application Suite (LAS) software v. 2.4.1; Roche Light cycler® 480 software v. 1.5.1.62; Typhoon FLA 9500 Control Software v. 1.0; BioRad Image Lab Software v. 6.1.0.
Data analysis	Agilent MassHunter Quantitative analysis v. B05.02 and B09.00; Agilent MassHunter Qualitative analysis v. B06.00 and B08.00; Image J v. 1.52r; Microsoft Excel v. 2016; GraphPad Prism v. 9.; Leica Application Suite (LAS) software v. 2.4.1; Roche Light cycler® 480 software v. 1.5.1.62

For manuscripts utilizing custom algorithms or software that are central to the research but not yet described in published literature, software must be made available to editors and reviewers. We strongly encourage code deposition in a community repository (e.g. GitHub). See the Nature Portfolio guidelines for submitting code & software for further information.

Data

Policy information about availability of data

All manuscripts must include a data availability statement. This statement should provide the following information, where applicable:

- Accession codes, unique identifiers, or web links for publicly available datasets
- A description of any restrictions on data availability
- For clinical datasets or third party data, please ensure that the statement adheres to our policy

All data are available from the corresponding authors upon request. Source data are provided with this paper.

Human research participants

Policy information about studies involving human research participants and Sex and Gender in Research.

Reporting on sex and gender	N/A
Population characteristics	N/A
Recruitment	N/A
Ethics oversight	N/A

Note that full information on the approval of the study protocol must also be provided in the manuscript.

Field-specific reporting

Please select the one below that is the best fit for your research. If you are not sure, read the appropriate sections before making your selection.

☒ Life sciences ☐ Behavioural & social sciences ☐ Ecological, evolutionary & environmental sciences

For a reference copy of the document with all sections, see nature.com/documents/nr-reporting-summary-flat.pdf

Life sciences study design

All studies must disclose on these points even when the disclosure is negative.

Sample size	Sample size was chosen such that statistical significance could be confidently established.
Data exclusions	No data was excluded.
Replication	Experiments were performed at least in triplicates, all results were reproducible.
Randomization	Randomization was not relevant to our study, since it does not involve different experimental groups where samples/organism/participants were allocated.
Blinding	Blinding is not relevant to our study, since it does not involve different experimental groups where samples/organism/participants were allocated.

Reporting for specific materials, systems and methods

We require information from authors about some types of materials, experimental systems and methods used in many studies. Here, indicate whether each material, system or method listed is relevant to your study. If you are not sure if a list item applies to your research, read the appropriate section before selecting a response.

Materials & experimental systems

n/a	Involved in the study	
☐	☒	Antibodies
☐	☒	Eukaryotic cell lines
☒	☐	Palaeontology and archaeology
☐	☒	Animals and other organisms
☒	☐	Clinical data
☒	☐	Dual use research of concern

Methods

n/a	Involved in the study	
☒	☐	ChIP-seq
☒	☐	Flow cytometry
☒	☐	MRI-based neuroimaging

Antibodies

Antibodies used	Mouse monoclonal anti-poly (ADP-ribose) antibody (Trevigen, 4335-MC-100, clone 10HA); anti-pan-ADP-ribose biding reagent (Merck, MABE1016); goat anti-mouse IgG-HRP conjugate (Dianova, 115-035-146); goat anti-rabbit IgG-HRP conjugate (Dianova, 111-035-144); mouse anti-ds DNA antibody [Abcam, ab27156, clone 35I9 DNA); mouse IgG control (Sigma-Aldrich I8765); mouse monoclonal anti-phospho-Histone H2A.X antibody (Millipore, 05 636-I, clone JBW301); goat anti-mouse IgG Alexa Fluor 488 conjugate (ThermoFisher, A-11029).
Validation	Mouse monoclonal anti-poly (ADP-ribose) antibody: PMID: 6206890; anti-pan-ADP-ribose biding reagent: https://

	www.merckmillipore.com/DE/de/product/Anti-pan-ADP-ribose-binding-reagent,MM_NF-MABE1016; mouse anti-ds DNA antibody : https://www.abcam.com/ds-dna-antibody-35i9-dna-bsa-and-azide-free-ab27156.html; monoclonal anti-phospho-Histone H2A.X antibody: https://www.merckmillipore.com/DE/de/product/Anti-phospho-Histone-H2A.X-Ser139-Antibody-clone-JBW301,MM_NF-05-636-I.

Eukaryotic cell lines

Policy information about cell lines and Sex and Gender in Research

Cell line source(s)	HEK293T (ATCC® CRL-11268™); mESC clones WT #4 (Meelad M Dawlaty, doi: 10.1016/j.devcel.2014.03.003); DIvA (Gaëlle Legube, doi: 10.1038/emboj.2010.38).
Authentication	None of the cell lines were authenticated.
Mycoplasma contamination	All cell lines were tested negative for mycomplasma contamination using Venor®GeM Mycoplasma detection kit (Minerva biolabs, Germany).
Commonly misidentified lines (See ICLAC register)	No misidentified cell lines were used in the study.

Animals and other research organisms

Policy information about studies involving animals; ARRIVE guidelines recommended for reporting animal research, and Sex and Gender in Research

Laboratory animals	Mouse organ tissues were obtained from 7-12 weeks old female and male C57BL/6J mice (Translational Animal Research Center, Mainz). Liver from adult male pig Sus scrofa domestica was from the University Medical Center Mainz.
Wild animals	The study did not involve wild animals.
Reporting on sex	Sex-based analyses was not performed
Field-collected samples	The study did not involve samples collected from the field.
Ethics oversight	No ethical approval or guidance was required since only mouse and pig organs obtained from collaborators were used for the study.

Note that full information on the approval of the study protocol must also be provided in the manuscript.
